# RNAseq based transcriptomics study of SMCs from carotid atherosclerotic plaque: BMP2 and IDs proteins are crucial regulators of plaque stability

**DOI:** 10.1038/s41598-017-03687-9

**Published:** 2017-06-14

**Authors:** Iraide Alloza, Haize Goikuria, Juan Luis Idro, Juan Carlos Triviño, José María Fernández Velasco, Elena Elizagaray, María García-Barcina, Genoveva Montoya-Murillo, Esther Sarasola, Reyes Vega Manrique, Maria del Mar Freijo, Koen Vandenbroeck

**Affiliations:** 1 Neurogenomiks, Neuroscience Department, Faculty of Medicine and Odontology, Basque Country University, Leioa, Spain; 2 Ikerbasque, Basque Foundation for Science, Bilbao, Spain; 3 ACHUCARRO, Basque Centre for Neuroscience, Zamudio, Spain; 4 Neurology Unit, Basurto University Hospital (Osakidetza/Basque Health Service), Bilbao, Spain; 5 Sistemas Genómicos, Valencia, Spain; 6 Radiodiagnostic Unit, Basurto University Hospital (Osakidetza/Basque Health Service), Bilbao, Spain; 7 Clinical Genetics Unit, Basurto University Hospital (Osakidetza/Basque Health Service), Bilbao, Spain; 8 Department of Methods and Experimental Psychology, Faculty of Psychology and Education, Deusto University, Bilbao, Spain; 9 Vascular Surgery and Angiology Unit, Basurto University Hospital (Osakidetza/Basque Health Service), Bilbao, Spain

## Abstract

Carotid artery atherosclerosis is a risk factor to develop cerebrovascular disease. Atheroma plaque can become instable and provoke a cerebrovascular event or else remain stable as asymptomatic type. The exact mechanism involved in plaque destabilization is not known but includes among other events smooth muscle cell (SMC) differentiation. The goal of this study was to perform thorough analysis of gene expression differences in SMCs isolated from carotid symptomatic versus asymptomatic plaques. Comparative transcriptomics analysis of SMCs based on RNAseq technology identified 67 significant differentially expressed genes and 143 significant differentially expressed isoforms in symptomatic SMCs compared with asymptomatic. 37 of top-scoring genes were further validated by digital PCR. Enrichment and network analysis shows that the gene expression pattern of SMCs from stable asymptomatic plaques is suggestive for an osteogenic phenotype, while that of SMCs from unstable symptomatic plaque correlates with a senescence-like phenotype. Osteogenic-like phenotype SMCs may positively affect carotid atheroma plaque through participation in plaque stabilization via bone formation processes. On the other hand, plaques containing senescence-like phenotype SMCs may be more prone to rupture. Our results substantiate an important role of SMCs in carotid atheroma plaque disruption.

## Introduction

Cerebrovascular disease is the third leading cause of disability and the second cause of death worldwide. Stenosis caused by atherosclerotic plaque formation in the carotid artery is one of the main factors to develop cerebrovascular symptoms^1^. However, certain patients develop carotid obstruction by plaque accumulation without presenting any associated symptoms. Therefore, atherosclerotic plaques can be defined as stable (asymptomatic, A) with a small fatty core and a thick fibrous cap or unstable (symptomatic, S) displaying a large fatty core and a thin fibrous cap prone to rupture^2^. Clinicians correlate plaque instability with the risk to develop cerebrovascular events^3^. The protocol of how to proceed with patients with carotid atherosclerotic plaque in asymptomatic patients may differ from hospital to hospital but generally patients with stenosis between 70% and 99% with an acceptable surgical risk, life expectancy with a good quality of life for at least 5 years and with no presence of severe disability would be indicated for carotid endarterectomy. This could be considered occasionally as an option to prevent potential future events since prevention is less costly than treating the complications of a potential cerebrovascular event. Recently, a debate has been initiated to assess the benefits of performing (or not) endarterectomy in asymptomatic patients^4^. New medical treatments with promising outcomes available nowadays in the market make the option of endarterectomy questionable for some patients. However, not all patients with asymptomatic carotid plaque would be benefiting from such treatments and would need anyhow to go through endarterectomy to increase their chances to evade cerebrovascular events. Some studies found that the effects of endarterectomy were correlated not only with lowering the risk of developing stroke^5^ but also with the improvement of cognitive performance, leading among others to increase in attention, verbal and visual memory, abstract thinking and verbal fluency^6–8^. Given these findings and the actual discussion about the potential benefits of endarterectomy in asymptomatic patients, the prediction of asymptomatic patients who would have high risk to develop unstable plaque will be crucial to help decide the best treatment option for the patient.

The precise mechanisms that cause destabilization and rupture of atherosclerotic plaque leading to an ischemic attack^9^ are still unknown. Atheroma carotid plaque consists mainly of macrophages/monocytes, endothelial cells and smooth muscle cells (SMCs). It is known that SMCs cells play a crucial role in the pathogenesis of atherosclerosis^10, 11^. One of the main events that occurs in this disease is the thickening of the intima caused by hyperplasia and migration of these cells from the media to the intima of the arterial wall^12^, initially promoted to repair vascular damage but which can then culminate in the development and progression of atherosclerotic plaque. SMCs cultures isolated from atherosclerotic lesions show a lower rate of proliferation and increased apoptosis/senescence than healthy SMCs^13^ indicating that the capacity of survival of these cells plays a role in the development of the atheromatous plaque.

The existing therapeutic available options for treating and preventing cerebrovascular disease have improved but are still limited^14^. For that reason, it would be especially useful to identify biological markers that would facilitate assessment of patients with high risk of developing cerebrovascular accidents. Despite intensive research^15, 16^, until now no serum biomarker has emerged successfully for clinical use in cerebrovascular disease. This could be due in part to the fact that stroke is a heterogeneous disease. In order to identify biomarkers that could be used in future prognostic/diagnostic approaches, efforts need to be done to better understand the molecular mechanisms underlying the disease. Analysis of carotid plaque tissue at a molecular level seems a suitable approach to unravel the mechanism/s involved in plaque disruption since atherosclerosis in the carotid artery is a risk factor for stroke. However, carotid atherosclerotic plaques are heterogeneous and composed of different cell types (endothelial cells, macrophages and smooth muscle cells). Previous studies analysing gene expression patterns in carotid plaques may not have had sufficient power to detect reproducible biomarkers due to cell type and content variability^17^. For that reason, in order to elucidate the mechanism/s involved in plaque destabilization, there is need to unravel the individual role that each cell type present in the plaque has. In an effort to clarify the role that SMCs play in plaque destabilization we carried out a comparative RNAseq based transcriptomics analysis in SMCs isolated from symptomatic plaques versus asymptomatic plaques. RNAseq technology is newly emerged omics approach that allows to achieve consistent high quality results. Up to now, no RNAseq based studies have been performed in human carotid cell types. The aim of this study was to perform a comparative analysis of the gene expression pattern in SMCs from stable and unstable carotid atheroma plaques.

## Results

### SMCs isolated from carotid atherosclerotic plaque express typical SMCs biomarkers

**Figure 1:**
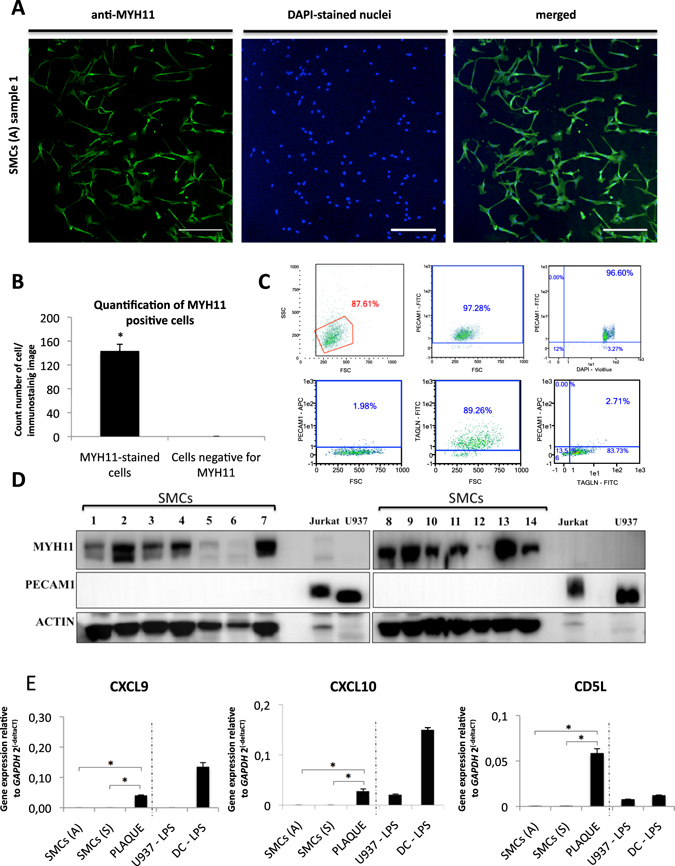
Quality control analysis. (**A**) Representative confocal images of MYH11-positive cells (left panel) and DAPI-stained cells (central panel) and merged (right panel). Magnification: the horizontal bars represent a scale of 250 μm; (**B**) Quantification of MYH11-positive cells versus total number of cells (DAPI-stained nuclei). Data are mean ± SD. *p < 0.05. (**C**) Flow cytometry graphs indicating expression of endothelial marker PECAM1 and SMCs marker TAGLN in SMC cultures (lower panels) and PECAM1 detection in U937 as positive control (upper panels). (**D**) Western blot detection in all our SMCs cultures of MYH11 and PECAM1 with ACTIN as loading control. Positive detection of PECAM1 and negative detection of MYH11 in Jurkat and U937 cells. (**E**) Gene expression quantification of macrophages markers (*CXCL9*, *CXCL10* and *CD5L*) performed by qPCR using RNA extracted from: (i) SMCs from asymptomatic patients (average of 7 samples); (ii) SMCs from symptomatic patients (average of 7 samples); (iii) atheroma plaque (average of 3 samples); (iv) monocyte cell line U937 treated with LPS; (v) CD14^+^ monocyte-derived dendritic cells treated with LPS.

Isolation and culture of primary vascular SMCs is considered an acceptable strategy to study the role of SMC in atherosclerosis^18^ and several isolation methods have been published, which can be grouped as either explant isolation or enzymatic digestion-based isolation. In our hands, the second option generated a greater amount of isolated cells. In particular, to isolate SMCs from carotid atheroma plaque we followed an enzymatic method described before with some modifications^19^ including two steps collagenase-I digestion protocol (3 hours and 16 hours). Cell viability after 3 or 16 hours digestion with collagenase was shown to be similar. Analysis of gene expression of SMCs specific marker *MYH11* in SMCs isolated after 3 hours of digestion or 16 hours showed no differences (Suppl. Fig. S1A). Digestion during 16 hours was found to yield higher numbers of cells than 3 hours digestion, and was therefore chosen as the standard protocol for generation of SMC cultures. Gene expression levels of passage 0 and 1 SMCs were compared to discard any effect of passage number on transcription of genes. Suppl. Fig. S1(A,B,C) shows no significant differences in gene expression of *MYH11*, *BMP2*, *ID1* and *ID4* genes in SMCs isolated from passage 0 and 1. To determine efficiency of isolation and purity of primary SMCs cultures, we performed several quality control tests. First, specific markers for smooth muscle cells were detected by immunostaining using specific antibodies for SMCs (i.e. α-MYH11)^20^ and endothelial cells (PECAM1- as negative control). This allowed us to examine the homogeneity of primary SMCs cultures, and was routinely done for all the SMCs cultures used in our study. Figure 1A shows representative images of one culture of SMCs isolated from carotid plaques with positive staining for MYH11, DAPI-stained nuclei and merged. Supplementary Fig. S2 shows another 3 different cultures stained with MYH11. The number of MYH11-positive cells in our cultures was identified by comparing the total number of cells (DAPI-stained nuclei) with the MYH11 positive stained cells (Fig. 1B) and this revealed absence of contaminating cells (i.e. fibroblast, endothelial cells, etc.). Second, MACS Quant flow cytometry analysis showed that only 2% of our cells were positive for PECAM1 detection (Fig. 1C), while 89% of cells were positive for SMCs marker TAGLN. Positive control of PECAM1 detection by flow cytometry was performed using U937 cells (Fig. 1C). Third, we analysed intracellular expression of SMCs specific protein (MYH11) and endothelial specific protein (PECAM1). Western blot analysis showed that isolated SMCs cultures all expressed MYH11, while they were negative for endothelial marker PECAM1. Fig. 1D shows protein identification by western blot using α-ACTIN, α-MYH11 and α-PECAM1 antibodies in cell lysates extracted from all SMCs cultures used for RNAseq analysis. Jurkat and U937 cell lysates were used as positive control of PECAM1 detection (Fig. 1D) Fourth, specific markers for macrophage-expressed *CXCL9* and *CXCL10* along with *CD5L*, the latter ﻿of which has been shown to be expressed in foam cells induced by oxLDL^21^, were tested in the 14 samples used for RNAseq analysis. RNA extracted from SMCs of asymptomatic and symptomatic patients showed no expression of *CXCL9*, *CXCL10* and *CD5L*. In contrast, carotid atheroma plaques, expected to contain macrophages, exhibited high expression of *CXCL9*, *CXCL10* and *CD5L*, similar to positive controls including human LPS-induced dendritic cells and LPS-induced U937 monocytic cell line (Fig. 1E). For these quality control analyses we routinely used passage 0–2 cell cultures.

### DEG and DEI analysis with transcriptomics data

To avoid potential transcriptional changes due to passage number, the RNA used for RNAseq analysis was in all cases extracted from smooth muscle cells at passage 0. RNA integrity (RIN) was assessed to ensure good quality of our RNA. All our RNA samples showed a RIN value between 9 and 10, which qualified them as suitable samples for RNAseq analysis^22^. A comparative transcriptomic analysis between the two groups, A and S, by applying RNAseq technology was performed to understand the role of SMCs in symptomatology of carotid atherosclerosis. Supplementary. Fig. S3 illustrates the flowchart of our RNAseq transcriptomics study. The number of replicates to be included per group (n = 7) was decided by the maximum availability of tissue obtained by a specific deadline taking into account the minimum of replicates recommended for this kind of studies as mentioned previously^23, 24^. The 14 generated libraries were sequenced on the Illumina Hiseq2500 platform. The data obtained with RNA sequencing was converted into quantitative gene expression levels with the aim of identification of differently expressed genes (DEG) and differently expressed isoforms (DEI) through comparison of A and S. We obtained between 85 and 87% mapped reads of total generated reads (Table 1), which was within the expected range of mapped reads onto the human genome (70–90%)^25^. GC content of all samples revealed a normal distribution (Supplementary Fig. S4) with peaks at the expected 40–60% range and distribution of duplicate content in our sequencing was found also to be within normal values (Supplementary Fig. S5). RNAseq data had to be normalized to eliminate any statistical deviation that could alter subsequent analysis. The main important deviations that we could encounter were the gene length, library size and GC content^26^ and to avoid such deviations normalization was performed using the DESeq2 proposed method^27^. Supplementary Fig. S6A and B show distribution of quantification before and after normalization. Principal component analysis (PCA) was performed on gene expression profiles using the method published before^28^ to identify possible outliers. PCA scores distinguished 2 outliers (A7 and S1), which would increase variability and decrease the power of our statistics, and were therefore removed from subsequent analysis. Dispersion adjustment illustrates how much the variance deviates from the mean and this was used to shrink the final estimates from the gene-wise estimates towards the fitted estimates, which improves gene expression analysis. Supplementary Fig. S7 shows the dispersion adjustment of data performed without the outliers.

**Table 1:** Summary of RNAseq mapping data. HQ, high quality.

Sample ID	Total reads	% HQ reads	% Properly pair reads	% Splice reads
1	61449044	86.11	72.15	26.38
2	76876864	86.51	71.27	25.33
3	67990002	85.99	70.8	25.14
4	67892558	86.08	70.74	26.91
5	71976326	86.78	72.31	26.78
6	74497186	82.21	72.82	23.61
7	72479266	87.66	71.85	27.01
8	70654274	84.75	72.09	24.79
9	73679062	87.23	71.45	27.43
10	70845658	86.19	71.21	25.85
11	72278906	86.59	72.24	25.42
12	72592062	86.26	70.91	27.36
13	80682878	85.84	73.04	26.56
14	72040720	86.47	70.94	26.49

In the differential expression analysis^29, 30^, we were able to identify 93 genes within the DEG analysis. A complete list of the 93 genes showing ﻿fold change (FC﻿) values ≥1.5(overexpressed in S vs A) and FC ≤ -1.5 (underexpressed in S vs A) with *P* value ≤0,05 (67 with *P* value (*P*_adj_) corrected by FDR ≤ 0,05)^31^ can be found in Supplementary Table S1. Among genes included in Supplementary Table S1, genes are associated with: *cell growth and senescence*, *bone metabolism*, *retinol metabolism*, *vascular disease*, *autophagy*, *immune system*, *muscle development*/*contraction*. Accurate isoform quantification remains a challenge due to high degree of overlapping between transcripts^32^. The variability in precision of isoform expression estimation across samples would be biased using traditional differential expression packages. For that reason, here we use the Bayesian approach Cufflink, which estimates transcript abundance based on how many reads support each transcript. DEI analysis identified 143 isoforms differently expressed between S and A. Supplementary Table S2 illustrates the isoforms with a FC value below −1.5 or higher than 1.5 and with *P*_adj_ ≤ 0,05^31^. Identified gene expression differences were also evaluated for gender differences since we had in our cohort only 2 woman in A and none in S, and no effect of gender was found within the genes listed in Supplementary Tables S1 and S2.

### Functional enrichment and network analysis

**Figure 2:**
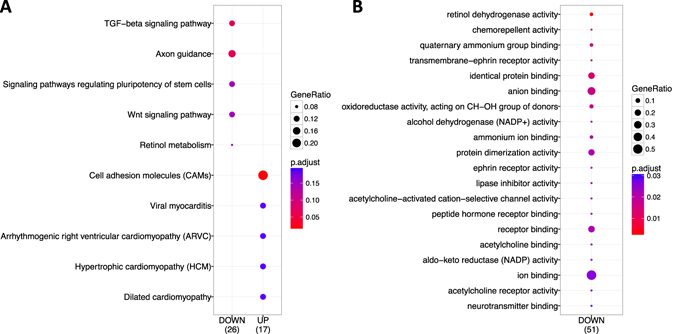
Comparative study between under-expressed and over-expressed genes using ClusterProfiler method. Comparative analysis based on KEGG (**A**). Comparative analysis based on Gene Ontology (**B**). GeneRatio corresponds to the number of genes from a specific category. Enrichment term is represented by coloured dots (red indicates high enrichment and blue indicates low enrichment).

**Figure 3:**
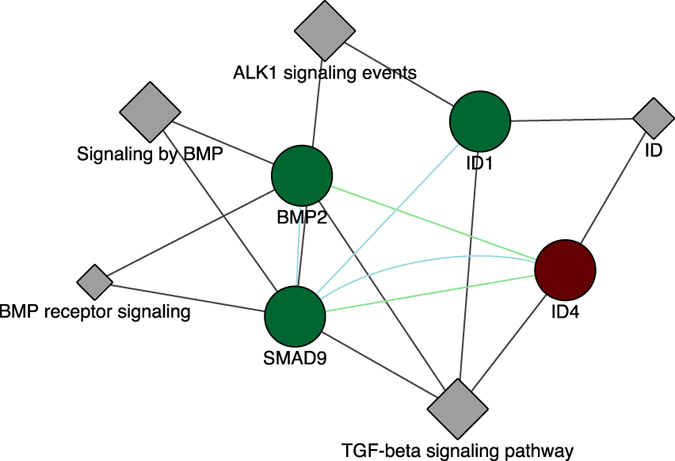
MCODE network clustering analysis. Illustration of the identified cluster by the network clustering algorithm MCODE showing the interaction between ID4, ID1, BMP2, SMAD9, TGF-beta signaling, BMP signalling and ALK1 signaling. The circles are genes and the rhomboids are protein domains. Green lines represent genetic interactions. Green circles indicate downregulated genes in S vs A and the purple circle indicates upregulated gene in S vs A. Blue lines represents pathways. Grey lines indicate gene-pathway associations.

Next, we performed functional and gene network analysis. Enrichment analysis allowing identification of combinations of significant annotations associated with the generated DEG and DEI lists was performed to identify functional relationships between the symptomatic and asymptomatic samples. Bioconductor-based enrichment analysis using as databases GO (Gene Ontology), KEGG (Kyoto Encyclopedia of Genes and Genome); reactome, DO (disease ontology), OMIM (Online Mendelian Inheritance in Man), and Cincinnati Children’s Hospital Medical centre^33–36^, generated a list of annotation groups which are significantly different between S and A. Supplementary Table S3 illustrates all annotations that included between 2 and 27 genes per group and that showed a q-value ≤ 0.05 generated from the DEG list, and Supplementary Table S4 illustrates the most significant annotations generated from DIE list. While Bioconductor-based analysis allowed us to identify common functional categories for a cluster of genes, we next compared these categories between the gene clusters by implementation of the algorithm proposed by the tool ClusterProfiler^37^. This tool, which is used for gene cluster comparison (i.e. downregulated and upregulated genes in S versus A), allowed us to visualize biological categories compared between gene clusters. The identified functional profiles based on KEGG, GO, Reactome and DO are illustrated in Fig. 2A,B, Supplementary Fig. S8A and B. KEGG-based comparison (Fig. 2A) is used to map RNAseq data for biological interpretation of higher-level systemic functions and identified TGF-β signalling pathway, axon guidance and cell adhesion molecules (CAMs) as those categories with higher GeneRatio number and lower *P*_adj_ value. Retinol dehydrogenase activity was identified in GO-based comparison, which defines functional gene classes and their relationships, as the category with the lowest *P*_adj_ (Fig. 2B). In addition, functional interaction network analysis using several sources of information such as protein interaction, genomics, pathways, etc. was performed using the program called GeneMANIA (Gene Multiple Association Network Integration Algorithm)^38^. Supplementary Fig S9 shows the functional network of genes obtained by GeneMANIA including clusters related with TFG-β signalling, axon guidance, cell adhesion molecules and ALK1 signalling. Subsequently, a further network analysis to identify a core network from the functional network generated with GeneMANIA was done with the method MCODE (Molecular Complex Detection)^39^. MCODE performs a network clustering step, which reduces the unconnected nodes in network clusters. Thus, the final cluster identified by MCODE was that with higher specificity and showed that the TGF-β, BMP and ALK1 signalling pathways arose as a functional process, which distinguish SMCs isolated from symptomatic or asymptomatic carotid plaques. Also notable is the identification in this network clustering analysis of 3 downregulated [*BMP2* (DEG: FC −1,9; *P*_adj_ 0,004/DEI: FC-4,27; *P*_adj_ 0,01)/ *SMAD9* (DEG: FC −1,51; *P*_adj_ 0,05)/*ID1* (DEG: FC −1,61; *P*_adj_ 0,03/DEI: FC-1,99; *P*_adj_ 0,01)] and one upregulated [*ID4* (DEG: FC 1,55; *P*_adj_ 0,09/DEI: FC 1,89; *P*_adj_ 0,01)] genes/isoforms in SMCs associated with plaque destabilization (Fig. 3).

### Confirmation of RNAseq identified biomarkers by digital PCR

Forty-one of the identified DEG RNAseq genes were selected for replication in 13 of the RNAseq-analysed samples (1 sample was not available) and for further validation in an additional cohort of 11 independent samples (4S and 7A) by digital qPCR (dPCR). The replication by dPCR showed very similar results to those found by RNAseq (Table 2) with similar fold-change directions (− or +) for 38 of these genes. Studies comparing RNAseq and qPCR data have indeed demonstrated very good correlation^40^ (Table 2). We could not detect 3 of the selected genes. In one case (*CHODL*) this may have been due to very low expression levels seen in the RNAseq data, which were undetectable by digital dPCR. In the other two cases (*SOCS3* and *TNFAIP8L*), where the expected expression levels should not have been low, technical issues relating to dPCR amplification & primer specificity may have been at stake. Combined analysis of the replication and validation datasets revealed significant fold changes coinciding with those of the original RNAseq with the exception of one gene (KCNE4; FC = 1.01). Thus, validation of 37 out of 38 genes attests to the accuracy of the RNAseq data. In the gene expression analysis done with only the 11 additional samples, the majority of genes apart of two (*HAPLN1 a*nd *TBX18*) showed similar fold change tendency. From those, 16 genes showed significant fold change differences, 7 genes showed tendency toward significance and 15 genes did not show any significant differences.

**Table 2:** Replication of 38 genes in RNAseq samples and validation in independent cohort.

Gene symbol	RNAseq (n = 14)		dPCR replication (n = 13)		dPCR validation (n = 11)		qPCR rep + val	
	FC	*P*_adj_	FC	*P*	FC	*P*	FC	*P*
PTHLH	−2,50	<0,0001	−1,32	0,001	−1,12	>0,1	−1,23	0,006
ANXA3	−2,23	<0,0001	1,08	0,02	−1,13	0,02	1,08	0,009
NPPC	−1,93	0,0038	−3,75	<0,0001	−10,16	0,0004	−4,54	<0,0001
ANXA10	−1,93	0,0035	−2,61	<0,0001	−1,63	0,09	−2,06	0,0003
DIRAS3	−1,93	0,0039	−2,80	0,0003	−1,29	0,08	−2,31	<0.0001
BMP2	−1,90	0,0049	−2,42	0,005	−3,87	0,08	−3,70	0,0018
ABCG2	−1,85	0,0104	−1,81	0,0008	−3,44	<0,0001	−2,09	<0,0001
TMTC2	−1,81	0,0159	−2,04	<0,0001	−1,73	0.03	−2,08	<0,0001
TMEM35	−1,81	0,0159	−2,37	0,03	−1,59	0,05	−2,13	0,0003
RGS2	−1,74	0,0188	−2,42	<0,0001	−1,71	0,08	−2,07	0,0006
ZSCAN31	−1,72	0,0107	−1,90	<0,0001	−1,82	0,03	−1,92	0,0001
MGST1	−1,72	0,0010	−1,33	0,007	−1,29	0,09	−1,32	<0,007
ESM1	−1,71	0,0329	−2,03	0,02	−1,99	0,05	−1,90	0,0017
KLN5	−1,70	0,0031	−1,49	0,0003	−1,39	0,04	−1,51	<0,0001
DHRS3	−1,70	0,0437	−2,96	<0,0001	−2,57	0,02	−3,14	<0,0001
FRMD3	−1,70	0,0419	−1,67	0,005	−1,85	0,02	−1,75	0,0005
ID1	−1,61	0,0368	−1,69	0,05	−5,01	0,009	−4,11	0,0008
EPHA4	−1,61	0,0470	−1,69	0,0002	−1,39	>0,1	−1,59	0,0003
HAPLN1	−1,58	0,0997	−2,16	0,0003	1,58	0,0038	−1,14	0,08
RDH10	−1,57	0,0585	−1,60	0,0002	−1,17	>0,1	−1,50	0,0019
EPHB2	−1,55	0,0278	−1,91	<0,0001	1,25	>0,1	−1,30	0,017
IL17RD	−1,54	0,0126	−1,28	0,01	−1,04	>0,1	−1,22	0,015
SMAD9	−1,52	0,0548	−1,68	0,003	−1,20	>0,1	−1,45	0,01
GULP1	−1,41	0,0471	−1,69	0,0003	−1,30	>0,1	−1,48	0,0032
DDR2	1,50	0,0219	1,48	0,001	1,01	>0,1	1,32	0,017
TBX18	1,51	0,0900	1,59	0,0003	−1,39	0,03	1,19	0,05
ADAMTS7	1,52	0,0224	1,96	<0,0001	1,04	>0,1	1,32	0,0017
PDK1	1,52	0,0339	1,71	<0,0001	1,17	>0,1	1,40	0,0002
PFKFB4	1,54	0,0389	1,33	0,003	1,27	>0,1	1,13	0,02
ID4	1,55	0,0900	1,73	0,001	1,01	>0,1	1,47	0,0037
SYNE3	1,60	0,0460	2,00	0,0019	1,01	>0,1	1,79	0,0067
CA12	1,72	0,0006	1,53	0,001	1,27	0,03	1,47	0,0002
MOCOS	1,75	0,0011	1,56	0,008	1,92	0,003	1,79	<0,0001
ITGA7	1,81	0,0051	3,05	<0,0001	1,58	0,09	2,06	<0,0001
ST6GALNAC5	1,83	0,0096	5,74	<0,0001	2,31	0,04	4,44	<0,0001
KCNE4	1,87	0,0031	1,33	0,02	1,01	>0,1	1,19	>0,1
MYO18B	1,70	0,0419	2,71	0,0002	1,06	>0,1	1,95	0,0021
C10orf10	1,94	0,0006	2,64	<0,0001	1,02	0,06	1,62	0,011

## Discussion

RNAseq technologies have been used as promising means for biomarker identification in several diseases lately^41, 42^. Since its discovery around 10 years ago, RNAseq has become an innovative tool for transcriptomics^43^. Stroke is a heterogeneous disease and the use of combination of biomarkers included in panels may enhance sensitivity and specificity of the applicability of biomarkers^17^. Pedrotty *et al*.^44^ reported the concept of multimarker panels for clinical use in cardiovascular disease. However, up to date only a few studies in human cerebrovascular research area have been performed by means of RNAseq technology^45, 46^, and these were performed in blood samples. In this study, RNAseq based transcriptomic data was generated from RNA extracted from SMCs isolated from carotid atheroma plaques from 7 A and 7 S patients with the aim of identifying candidate genes and pathways that could discriminate the two groups studied, S and A. We ensured high quality control and purity of the SMC isolates. 16 hours collagenase digestion showed similar expression of MYH11 as 3 hours. Therefore, we expected that our results have not been significantly affected by 16 hours digestion protocol. In addition, the use of passage 0 SMCs in our study should track gene expression patterns closest to physiological conditions in unperturbed plaques. Using passage 0 would also avoid any effect due to phenotype changes that SMCs could suffer during passages (i.e. senescence observed from passage 8)^47^. We identified genes/transcripts candidates for panel/s, which have a potential to classify the two studied groups, A and S (Suppl. Tables S1 and S2).

Transforming growth factor beta (TGF-β) signalling was identified in this study as a crucial pathway in symptomatology of carotid atherosclerosis at the smooth muscle cell level. TGF-β signalling emerged initially in the functional enrichment analysis performed (Fig. 2A) and the subsequent MCODE network clustering identified TFG-β signaling together with BMP and ALK1 signaling in the cluster with highly interacting nodes (Fig. 3). TGF-β superfamily members are involved in several processes including cell proliferation, migration, extracellular matrix production, bone morphogenesis, etc. and can be classified into two groups: TGF-β/activin/nodal and BMP/GDF^48^. Bone morphogenetic proteins (BMPs) are growth factors of the TGF-β family and Smads proteins (R-Smad, Co-Smad, I-Smads) are signal transducers that participate in the TGF-β signalling pathway. The subfamily of Smads called R-Smads includes several proteins of which some are involved in the BMP signalling branch (i.e. Smad1, Smad5 and Smad9)^49^. Some BMPs proteins have osteogenic activity capacity; in particular BMP2, which belongs to the BMPs that play a role in ossification^50^. It has also been reported that BMP2 inhibits SMCs cell growth and migration, promoting a contractile phenotype^51^, which is observed in normal vessels. High levels of BMP2 in blood have been recently associated with plaque progression and calcification in coronary atherosclerosis^52^. We found significantly lower levels of expression of *BMP2* in our symptomatic SMCs compared with asymptomatic, which is indicative for a beneficial role of *BMP2* in atheroma plaque stability both by contributing to the stabilization through bone morphogenesis and by promoting a protective phenotype, which may be called an osteogenic phenotype. BMP2 plays a role in ALK1 signaling, in which ALK1 (activin receptor-like kinase-1) is a type I receptor of TGF-β that is regulated by BMP2 expression in vascular cells enhancing vascular cell proliferation^53^. Also, the BMP-activated SMAD9 gene^54^ showed lower levels in S vs A (Suppl. Tables S1, S2). Id genes (*Id1*, *Id2*, *Id3* and *Id4*) have been associated with regulation of several cellular processes such as cell growth, senescence, angiogenesis and apoptosis. In particular, *Id1*, *Id2* and *Id3* genes have been identified as early response genes in BMP signalling that can bind Smad proteins^55^. Interestingly, BMP2 induced the expression of *Id1* in the mouse myoblast cell line C2C12^56^ and upregulation of the *Id1* gene is known to delay senescence in endothelial cells^57, 58^. Similarly to endothelial cells, our results suggest that *Id1* expression in SMCs exhibited a role as anti-senescence factor, which may be associated with plaque stability. While Id1/Id2/Id3 are expressed in multiple tissues, Id4 has been reported to be expressed in neuronal tissues and intestinal embryonic epithelium^59, 60^. We found that *Id4* expression levels in all our SMCs samples were not lower but even higher than the other Id genes. Also upon comparison of *Id4* levels between S and A, we found that *Id4* expression was higher in symptomatic SMCs samples, suggestive for senescence-like phenotype. This is also consistent with the observation the overexpression of Id4 in astrocyte-derive cell line induces apoptotic cell death^61^.

As TGF-beta signalling pathway regulates a wide range of cellular processes through diverse effects, such as modulation of angiogenesis by affecting cell proliferation, migration and differentiation, it has been considered a target for cardiovascular diseases^62^. Several cardiovascular disorders have been linked to TGF-beta signalling pathways: (i) in hemorrhagic telangiectasia type I (HHT-1), lower endoglin expression in ECs was found to be associated with the disease^63^, and endoglin underexpression was thought to lead to the reduced levels of TGF-β secretion found in HHT-1 patients; (ii) Loeys-Dietz syndrome is characterized by mutations in TbRi or TbRII genes, which are known to affect the TGF-beta signalling pathway^64^; (iii) mutations in BMP receptor BMPRII in SMCs have been associated with pulmonary arterial hypertension (PAH)^65^; (iv) it has also been described that the TGF-β signalling pathway is altered in cardiac remodelling and hypertrophy^66^. However, the precise role of the TGF-beta pathway in cardiovascular diseases is dependent on stage of disease as well as on the specific location of its activity implying that not only tissue/organ-type (i.e. aortic artery, carotid artery, heart, etc.) but as well cell types have to be considered before making general conclusions.

Other genes identified in our differential expression analysis include those related to bone mineralization matter, such as carbonic anhydrase 12 (CA12), parathyroid hormone-like hormone (PTHLH) and natriuretic peptide C (NPPC). *CA12* showed higher expression in SMCs from S vs A (FC 1,72 (*P*_adj_ = 0,0006)) in our study and although during initial phase of atherosclerosis bone resorption could be beneficial^67^, in later stages, such as in advanced plaques from symptomatic patients, bone resorption may affect the plaque destabilization process. Conversely, PTHLH was found to be downregulated in SMCs from S vs A (FC −2,5 (*P*_adj_ = 0,0000)). PTHLH has been shown to increase bone formation and decrease bone resorption^68^. Similarly, NPPC (FC −1,93 (*P*_*adj*_ = 0,0038)) was found to be downregulated in S vs A and is as well considered a positive regulator of bone formation^69^. Thus, this finding may reinforce our hypothesis that SMCs from A benefit from bone mineralization processes. Thus, RNAseq transcriptomics has revealed significant differences in gene expression profiles between SMCs from A and S carotid plaques and this has provided clues as to the role SMCs may play in carotid atheroma plaque destabilization. In summary, taken together our gene expression profiling data and combined analysis, SMCs isolated from asymptomatic patients may exhibit a calcified/osteogenic-like phenotype, while SMCs from symptomatic patients exhibit a senescence-like phenotype.

## Materials and Methods

### Ethics statement

This study was approved by the local ethical committee (Ethical committee of clinical research, Basurto Hospital) and all carotid atheroma plaques were collected from patients who had signed written informed consent. This research was performed in agreement with the principles outlined in the Declaration of Helsinki.

### Patient selection

Patients who underwent carotid endarterectomy at Basurto University Hospital were selected to be included in the current study on the bases of clinical parameters (age, symptoms, no other clinical manifestations). Degree of stenosis was evaluated with carotid cervical Eco-Doppler ultrasound and tomographic angiography according to established criteria^70^. Symptomatic patients were identified as those with >70% stenosis and presenting symptoms of transient ischemic attack or ipsilateral stroke within the past 6 months, while asymptomatic patients were those with stenosis >80% without any presence of cerebrovascular disease. All patients were evaluated before and after the operation by a specialized neuropsychologist. Brain MRI was performed in all patients before and after the surgery to monitor potential complications. Only patients who fulfil all the required parameters were finally included in the study (7A vs 7S). Carotid tissue sample was collected after surgery and transported immediately to the lab for cell isolation.

### Experimental approach to distinguish SMCs isolated from symptomatic and asymptomatic carotid plaques by RNAseq technology

The study was designed with maximum power to detect DEG and DEI as well as to perform complementary functional enrichment and network analysis. The two main factors to take into account in RNAseq transcriptomics studies are the number of replicates and the sequencing depth. Thus, RNA sequencing was performed with a depth to obtain around 60 millions reads per sample, which allowed us to reach reliable detection power of differential expression^71^. Sequencing at this depth would allow to identify differential expression with maximum power in 5 replicates, even of those genes with low expression^24^. Also, variability in RNAseq analysis is reduced when studying a specific cell type (i.e. SMC) compared with studying the full atheroma plaque, which is heterogeneous and composed of varying numbers of endothelial cells, SMCs and macrophages. Accordingly, to study the role of SMCs in the development of unstable carotid atherosclerosis, we selected 14 patients (7 symptomatic and 7 asymptomatic) who were proposed for carotid endarterectomy by the committee of medical experts from Basurto University Hospital, who evaluated the clinical data of patients (Table 3). MRI and cervical duplex was performed in all patients by the radiology service at the hospital. Patients with >70% stenosis and diagnosed cerebrovascular event symptomatic (S) and patients with >80% stenosis and no events asymptomatic (A) were included in the study. Other required parameters than symptomatic (S) and asymptomatic (A) for selection of eligible patients to be included in this study were defined as: (1) age (70 years ±12); (2) no other medical conditions and (3) no previous contralateral endarterectomy, (4) no other cause of stroke, such us cardioembolic. During the recruitment period several patients were excluded from the study because they presented at least one of the following problems: (1) patients showing sign of dementia according to the minimental state examination test (MMSE); (2) patients with psychological alterations due to drugs or cerebral surgery intervention; (3) patients who presented medical or surgery complications that may alter the intellectual capacity and/or the neuroimaging test and (4) patients who were not able to perform the neuropsychological test due to difficulty of communication (i.e. vision impairment).

**Table 3:** Demographic data for Basurto University Hospital cohort of samples.

Patient characteristics	All	Symptomatic	Asymptomatic
Number, n	14	7	7
Years	68	68 ± 8	68 ± 8
Sex M/F, n	11/3	4/3	7/0
Treatment with statins	14	7	7
**Risks Factors (%**)			
Diabetes Mellitus	100	3 (43%)	4 (57%)
Dyslipidemia	100	7 (100%)	7 (100%)
Arterial hypertension	100	7 (100%)	7 (100%)
Tobacco	100	4 (57%)	3 (43%)

### Smooth muscle cells culture and RNA purification

Fresh carotid artery tissue samples from symptomatic and asymptomatic patients were processed and cut in 2–3 mm size fragments, which were digested with collagenase type I (Life Technologies, Carlsbad, CA, USA) to further isolate and culture SMCs. First, we performed 3 hours digestion at 300 U/mL concentration of collagenase type I. Second, digested tissue was submitted to overnight digestion with 220 U/mL collagenase type I. Digested tissue was filtered by a 100 µm nylon Falcon™ Cell Strainer for removing undigested material, and cells were plated in specific medium for human SMC growth called M231 (Life Technologies, Carlsbad, CA, USA) supplemented SMCs growth factors such us 20 ng/ml IGF-1, 2 ng/ml EGFB, 0,5 ng/ml EGF, 5 ng/ml heparin, 0,2 μg/ml BSA and 5% newborn calf serum (Sigma, St Louis, MO, USA). Second digestion yielded higher amount of cells. RNA used for RNAseq was extracted from cells from passage 0. RNA used for quality control analysis was extracted from SMCs isolated after 3 or 16 hours collagenase digestion and from passages 0 and 1. Quality control of pure SMCs culture was performed by identification of the presence of SMC-specific marker (MYH11) and absence of endothelial marker (PECAM1) and macrophage markers (CXCL9, CXCL10 and CD5L). As positive control for CXCL9 and CXCL9 we used RNA extracted from monocytic cell line U937 induced with LPS (100 ng/ml) during 6 hours and CD14^+^ monocyte-derived dendritic cells treated with LPS (100 ng/ml) for 6 hours. Extraction and purification of RNA from SMCs was performed with TRIzol^TM^ reagent according to manufacture’s instructions. RNA was then purified with RNeasy Total RNA Isolation Kit (QIAGEN, Valencia, CA, USA) following the instructions recommended by the manufacturer. RNA integrity (RIN > 9) was analysed by using RNA 6000 Nano Chips on the Bioanalyzer Agilent 2100 (Agilent Technologies, Palo Alto, CA, USA).

### Western blot

Cell lysis was performed with RIPA lysis buffer (150 mM TrisHCL, 150 mM NaCl, 0.5% Deoxycholate, 0.1% SDS, 1% NP-40) for 30 min at 4 °C followed by centrifugation at 20,000*xg* for 10 min. Cell lysates were submitted to 6%- or 12% SDS-PAGE gels. Proteins were transferred to PVDF membrane. Inmunodetections were done with following primary antibodies: anti-smooth muscle myosin heavy chain 11 (ab82541, Abcam, Cambridge, UK) and anti-CD31 antibody (PECAM1-EPR3094 ProteinTech, Chicago, USA). Proteins were detected with Immobilon™ Western Chemiluminescence HRP Substrate detection reagent (Millipore, Billerica, MA, USA) and were visualized with the ChemiDoc™XRS Imaging System (Bio-Rad, Richmond, CA, USA). Anti-ACTIN antibody (A2066, Sigma-Aldrich, St Louis, MO, USA) was used as housekeeping for normalization. Data analysis was done using Image Lab™ Software (Bio-Rad, Richmond, CA, USA).

### Microscopy

Cells were grown on 24 well plates with round glass coverslips (Thermo Scientific) followed by fixation in ice-cold methanol. Afterwards, samples were blocked in PBS/3% w/v BSA for 30 min and stained with anti-smooth muscle myosin heavy chain 11 antibody for 1 h at room temperature. Cells were washed with PBS and incubated with Alexa Fluor^®^488 goat anti-rabbit IgG (H + L) secondary antibody for 45 min at room temperature in darkness and DNA was counterstained with 4′,6-diamino-2-fenilindol (DAPI). After three washes coverslips were mounted in Fluoromount™ Aqueous Mounting Medium (Sigma-Aldrich, St Louis, MO, USA). Image acquisition was performed with Leica TCS STED CW SP8 super-resolution microscope with a 40x or 10x lens and recording optical sections every 0.3 µm. Image analysis was done using ImageJ software (National Institute of Health, USA).

### Flow cytometry

SMCs cultures were fixed in 4% formaldehyde, permeabilized with saponin and blocked with bovine serum albumin. Cells were incubated with primary antibody anti-PECAM1 or anti-TAGLN for 1 hour at room temperature. Secondary antibody incubation was performed with Alexa Fluor^®^488 goat anti-mouse antibody. Washed cells were incubated in 5 mM EDTA and 0.5% BSA. Cells were analysed using a Miltenyi MACS Quant flow cytometer (Miltenyi Biotec, Bergisch Gladbach, Germany).

### RNA library assembly

Ribosomal RNA removal was performed with the Ribo-Zero rRNA kit removal kit. Generation of libraries was performed with the TruSeq Stranded Total RNA library Prep kit following manufacture’s recommendations. We started from 2 μg of total RNA (RIN > 9) libraries, which were sequenced using a HiSeq2000 instrument (Illumina Inc, San Diego, CA, USA). Sequencing readings were paired-end with a length of about 100 bp reading performed in 14 samples. The estimated coverage was around 60 million reads per sample (3 lanes). Library generation and RNA sequencing was done at Sistemas Genómicos S.L. (Valencia, Spain) following manufacturer´s instructions.

### RNA transcriptomics analysis

The quality control of the raw data was performed using the FastQC tool. Then, the raw paired-end reads were mapped against the human genome provided by Ensembl database (version GRchr37/hg19) using tophat2 algorithm^72^. Insufficient quality reads (phred score < 10) were eliminated using the Picard Tools software (version 1.129). In this step, we assessed the GC distribution (i.e. the proportion of guanine and cytosine bp along the reads), which should have a desired distribution between 40–60%. Second, distribution of duplicates (quality of sequencing indicator) were evaluated to confirm that our sequencing contained small proportion of duplicates. Gene predictions were estimated using the Cufflinks method^73^ and the expression levels were calculated using the HT Seq software (version 0.6.0, http://www-huber.embl.de/users/anders/HTSeq/). This method employs unique reads for the estimation of gene expression and eliminates the multimapped reads. Differential expression analysis between conditions was assessed using DESeq2 method^30^ (version 3.4). Finally, we selected differentially expressed genes with a *P* value adjusted by FDR^31^ < 0.05 and a fold change of at least 1.5. The DEG analysis between S and A was done by using statistical packages designed by Python and R. Using the DESseq2 algorithm^30^ applying a differential negative binomial distribution for the statistics significance^31^ we identified genes and isoforms differentially expressed. We considered as differently expressed genes or isoforms^30^ those with a FC value below −1.5 or higher than 1.5 and with *P* value (*P*_adj_) corrected by FDR ≤ 0,05^31^ to avoid identification of false positives across the differential expression data.

### Gene Set functional enrichment analysis and network analysis

Differentially expressed sets were processed using ClusterProfiler^37^, a bioconductor package, to search for biological processes involved in plaque unstability. This tool screens for genes in specific databases (i.e. Gene Ontology – GO, Kyoto Encyclopedia of Genes and Genomes – KEGG, DRUG, etc) to evaluate biological annotations that rise as over-represented with respect to the whole genome. Gene networks were generated using GeneMANIA software (http://www.genemania.org/). GeneMANIA uses functional interconnections among genes from published data to generate a global view of interactions of genes. MCODE tool (http://apps.cytoscape.org/apps/mcode) was used to identified highly interconnected clusters in a network.

### Digital PCR on Fluidigm Biomark platform

Assays for 41 genes identified in our RNAseq DEG analysis and 4 housekeeping genes chosen from our own DEG as those showing no variability between samples (*G3BP2*, *MKLN1*, *EML3* and *ADCK5*) were use for validation. RNA was converted into cDNA using the AffinityScript Multiple Temperature cDNA Synthesis kit (Agilent, Technologies, Santa Clara, CA, USA) following manufacture’s instructions. Gene expression analysis was performed with the Fluidigm Biomark 48.48 dynamic arrays (Fluidigm Corp., South San Francisco, CA, USA) and pre-designed PrimeTime qPCR assays (IDT, Leuven, Belgium) in 24 samples in duplicates (13 samples corresponding to RNAseq samples and 11 extra samples). Patient characteristics for the additional 11 samples are giving in Supplementary Table S5. Gene expression levels of housekeeping genes were used as endogenous control to normalize our samples. Relative expression was calculated using the ∆∆Ct method^74^. The statistical significance of gene expression levels between S and A was analysed with the GraphPad Prism 5 (GraphPad Software, La Jolla, CA, USA) using the non-parametric Mann-Whitney U-test (one-tailed) and the level of significance was defined at *P* < 0.05. All PrimeTime qPCR assays showed amplification efficiencies close to 100%.

### Real Time qPCR

SYBRgreen technology was used for gene expression of *CXCL9*, *CXCL10*, *CD5L* and *BMP2* for quality control of SMCs purity. PrimeTime qPCR primes (IDT, Leuven, Belgium) were used and GAPDH was used as housekeeping gene for normalization. Gene expression was detected with the ABI7500Fast equipment (Life Technologies, Carlsbad, CA, USA). The analysis was performed using the ∆Ct method and expressed as 2^(−∆Ct)^. The statistical significance was calculated as described above using GraphPad Prism 5.

## Electronic supplementary material

Supplementary information
