# Supplementary material for: RNAseq based transcriptomics study of SMCs from carotid atherosclerotic plaque: BMP2 and IDs proteins are crucial regulators of plaque stability
Source: Sci Rep. 2017 Jun 14;7:3470. doi: 10.1038/s41598-017-03687-9 (PMC5471186; doi:10.1038/s41598-017-03687-9)
Supplement: Supplementary file 1 — Supplementary information [file 41598_2017_3687_MOESM1_ESM.pdf]

Supplementary Information

RNAseq based transcriptomics study of SMCs from carotid atherosclerotic plaque: BMP2 and IDs proteins are crucial regulators of plaque stability

Iraide Alloza[1,2,3*], Haize Goikuria[1,3], Juan Luis Idro[4], Juan Carlos Triviño[5], José María Fernandez Velasco[4], Elena Elizagaray[6], Mª García Barcina[7], Genoveva Montoya[8], Esther Sarasola[7], Reyes Vega[9], Mª del Mar Freijo[4], Koen Vandenbroeck[1,2,3]

Affiliation(s):

[1]Neurogenomiks, Neuroscience Department, Faculty of Medicine and Odontology, Basque Country University, Leioa, Spain.

[2]IKERBASQUE, Basque Foundation for Science, Bilbao, Spain.

[3]ACHUCARRO, Basque Centre for Neuroscience, Zamudio, Spain.

[4]Neurology Unit, Basurto University Hospital, Bilbao, Spain.

[5]Sistemas Genómicos, Valencia, Spain.

[6]Radiodiagnostic Unit, Basurto University Hospital, Bilbao, Spain.

[7]Clinical genetics Unit, Basurto University Hospital, Bilbao, Spain.

[8]Department of Methods and Experimental Psychology, Faculty of Psychology and Education, Deusto University, Bilbao, Spain.

[9]Vascular surgery and angiology, Basurto University Hospital, Bilbao, Spain.

*Corresponding author: Iraide Alloza PhD, Neurogenomiks Group, Neuroscience Department, Faculty of Medicine and Odontology, University of Basque Country (UPV/EHU), Edif 205, floor -1, Bizkaia Technological Park, 48170 Zamudio (Bizkaia), Spain, email: iraide.alloza@ehu.es, phone: +34 946018292, fax: +34 946018289

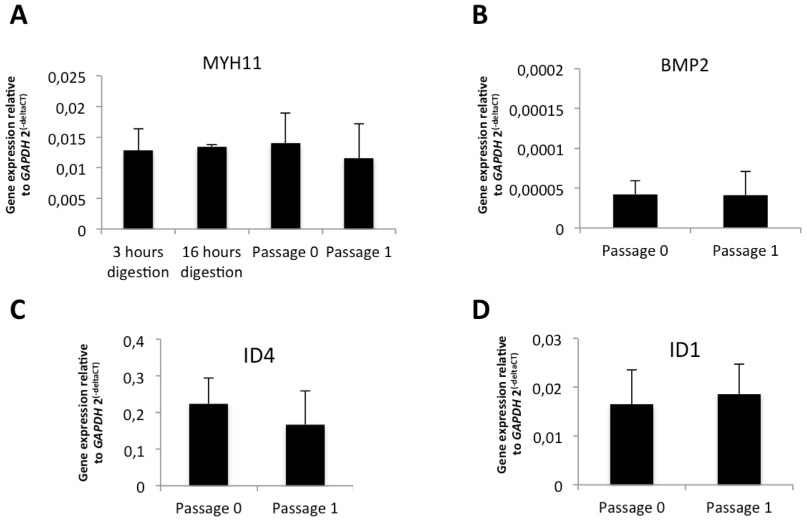

Supplementary Figure S1. (A) Differential gene expression analysis of *MYH11* in SMCs isolated after 3 and 16 hours of collagenase digestion and in SMCs from passage 0 and 1. (B) Differential gene expression of *BMP2* in passage 0 vs passage 1. (C) Differential gene expression of *ID4* in passage 0 vs passage 1. (D) Differential gene expression of *ID1* in SMCs from passage 0 and passage 1. GAPDH was used as housekeeping gene for normalization. Figure shows a representative analysis of gene expression levels in two different SCMs samples in each condition with 4 performed replicates to illustrate quality control (MYH11).

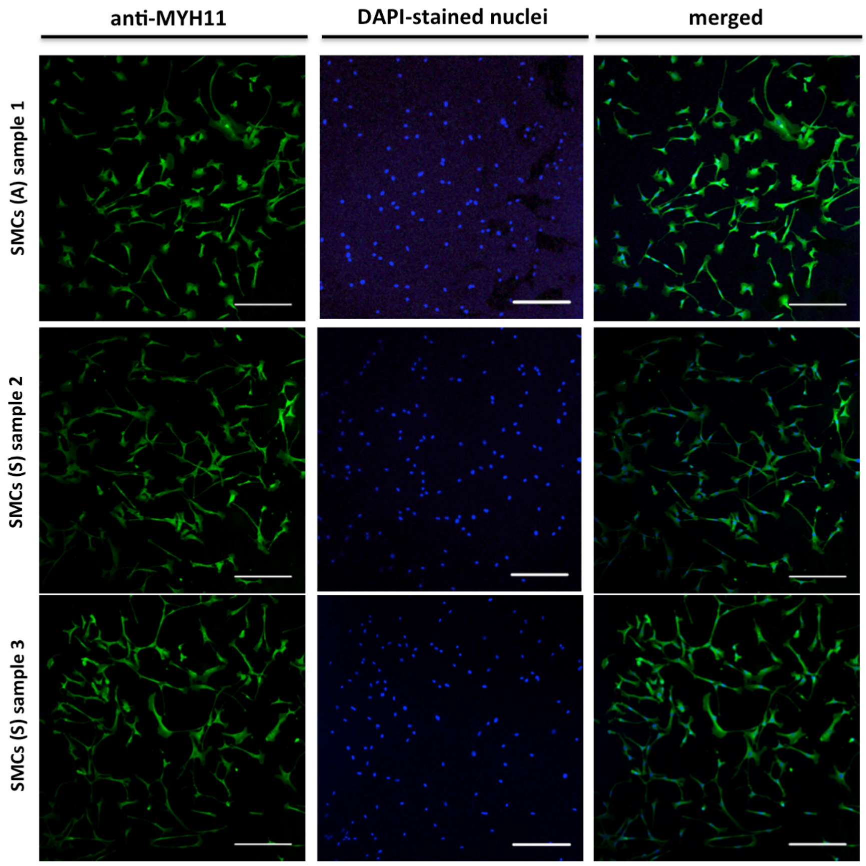

Supplementary Figure S2. Immunolocalization of MYH11 in primary carotid SMCs. Confocal immunostaining of 3 different cultures of SMCs as representative examples. Panels on the left show immunostaining of MYH11, central panels show DAPI-stained nuclei and right panels show immunostaining merged with DAPI staining. Magnification: the horizontal bars represent a scale of 250 μm.

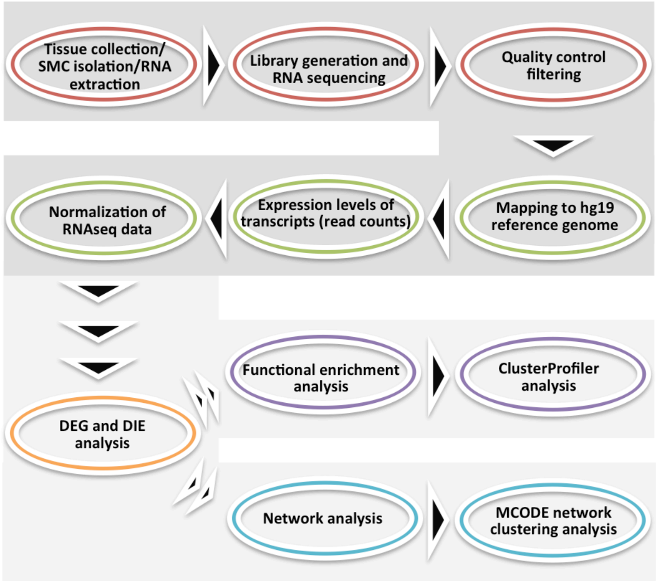

Supplementary Figure S3. Flowchart for the design of the RNAseq transcriptomics analysis to identify gene expression differences between SMCs from A and S carotid plaques.

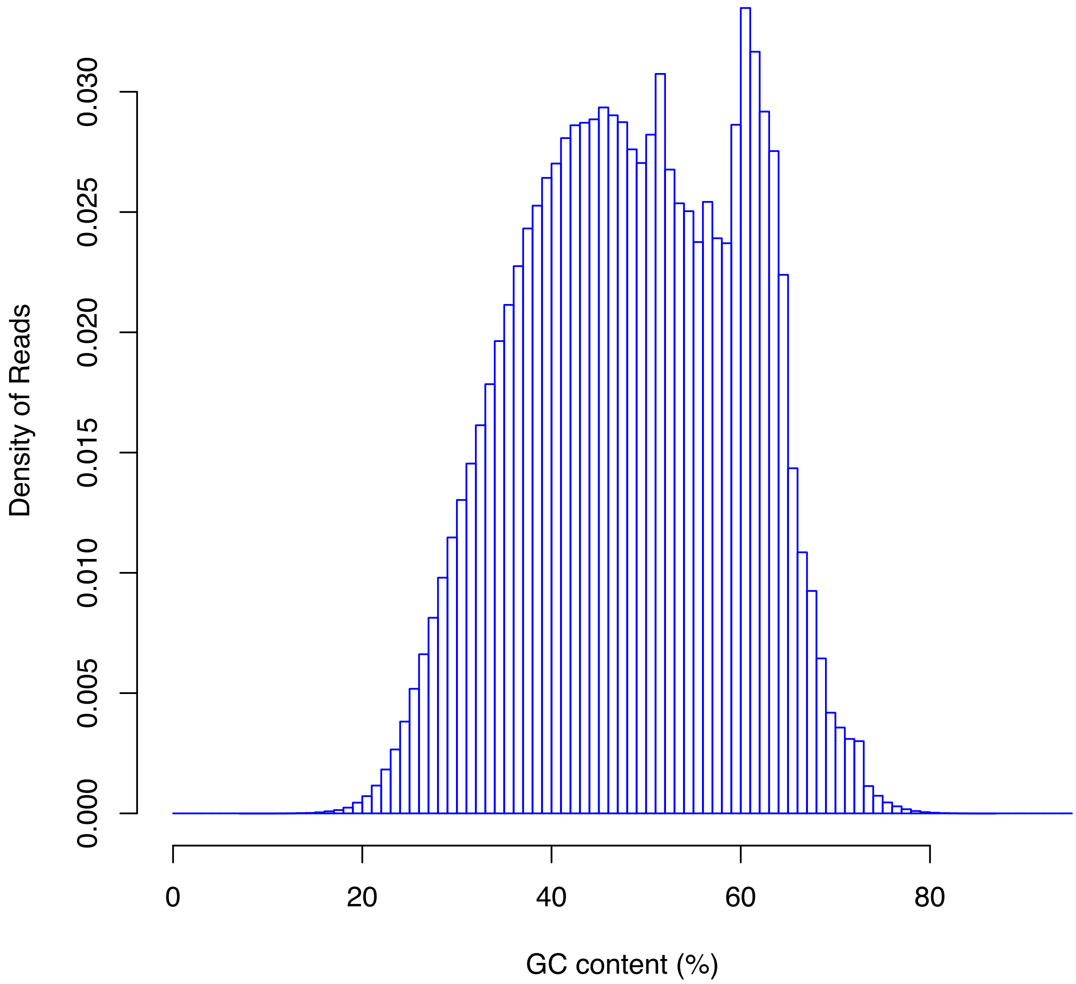

Supplementary Figure S4. Distribution of GC content. All the samples followed a normal distribution of GC content, expected to be between 45-55 %.

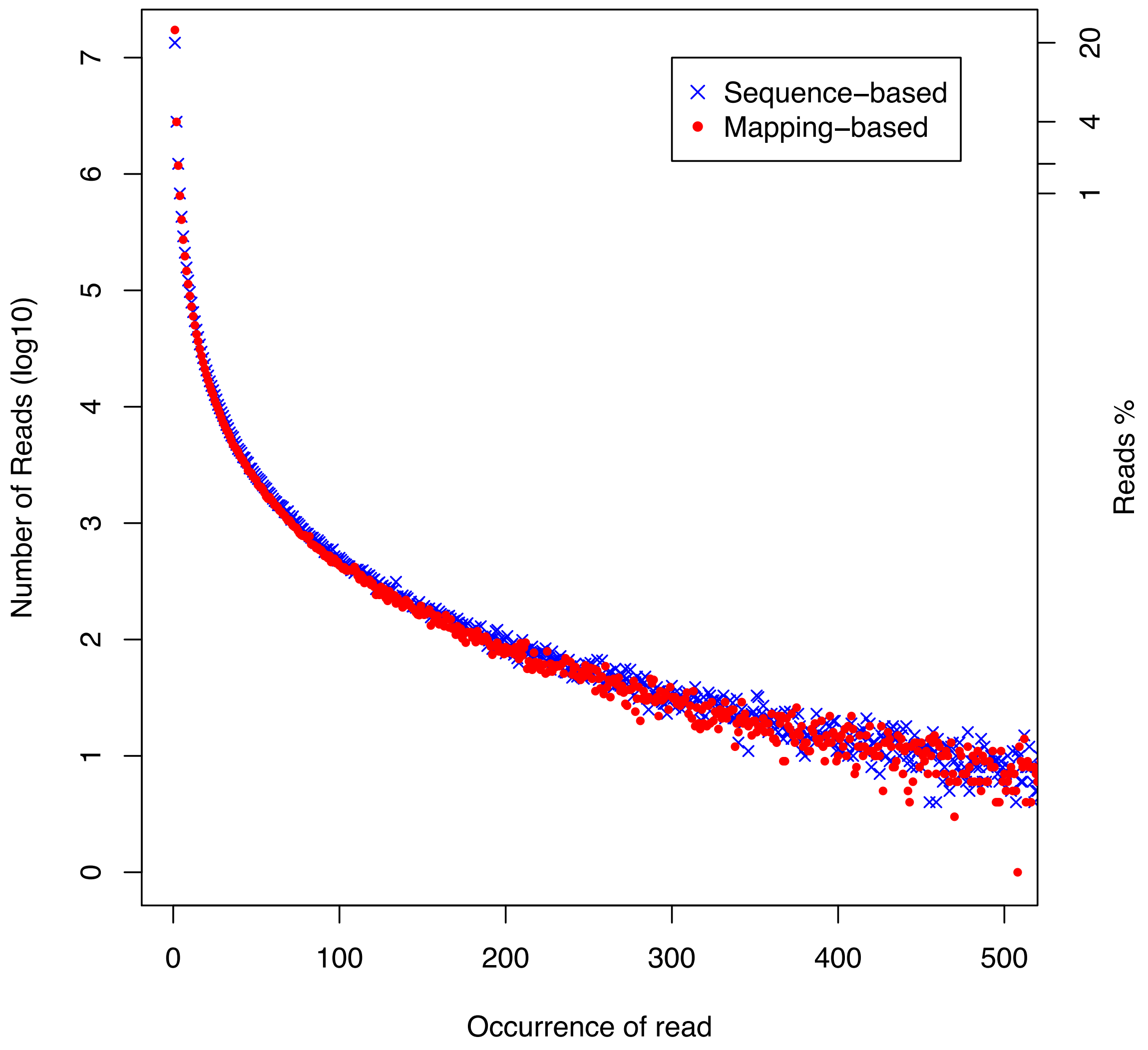

Supplementary Figure S5. Distribution of duplicates. Only a very small fraction of reads showed a small number of duplicates.

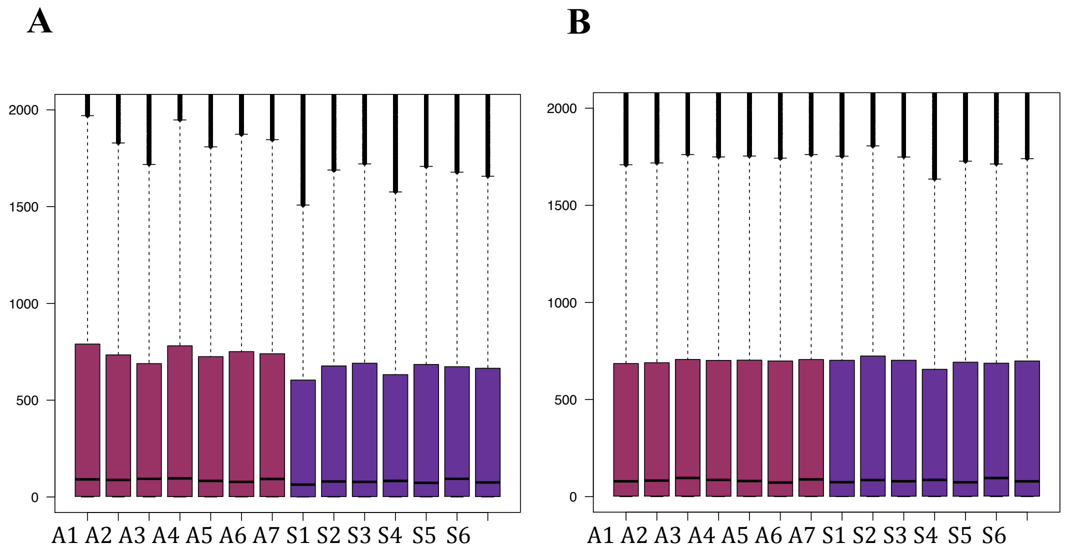

Supplementary Figure S6. (A, B) Normalization by DESeq2. Distribution of gene quantification before normalization (A) and after normalization (B).

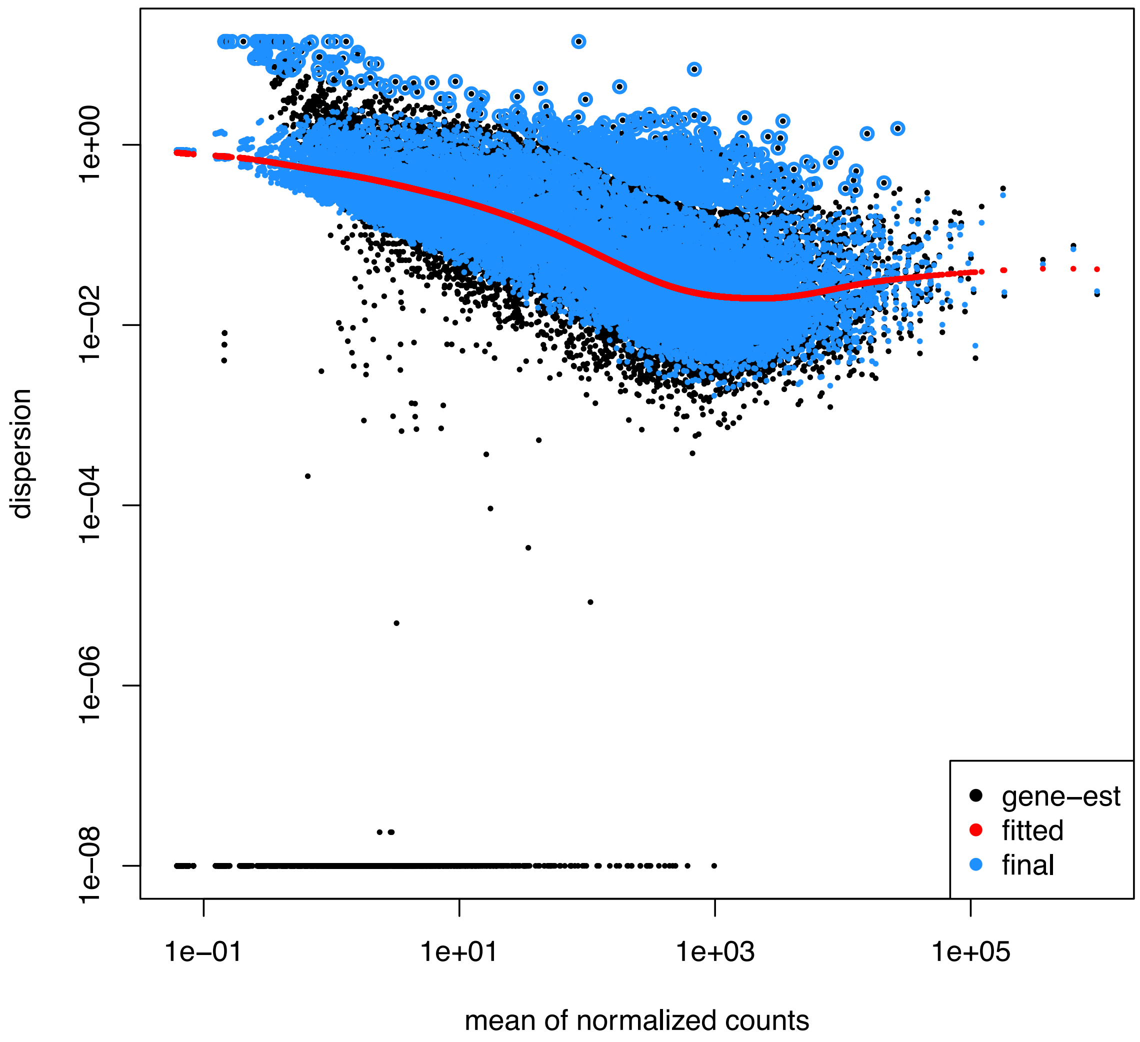

Supplementary Figure S7. Dispersion adjusment plot. The dispersion adjustment plot displays the gene-wise estimate (black), the fitted values representing the relationship between the dispersion and the normalized counts (red), the final estimates (blue) and the dispersion outliers values with high gene-wise dispersion, which were not adjusted (blue-circle).

A

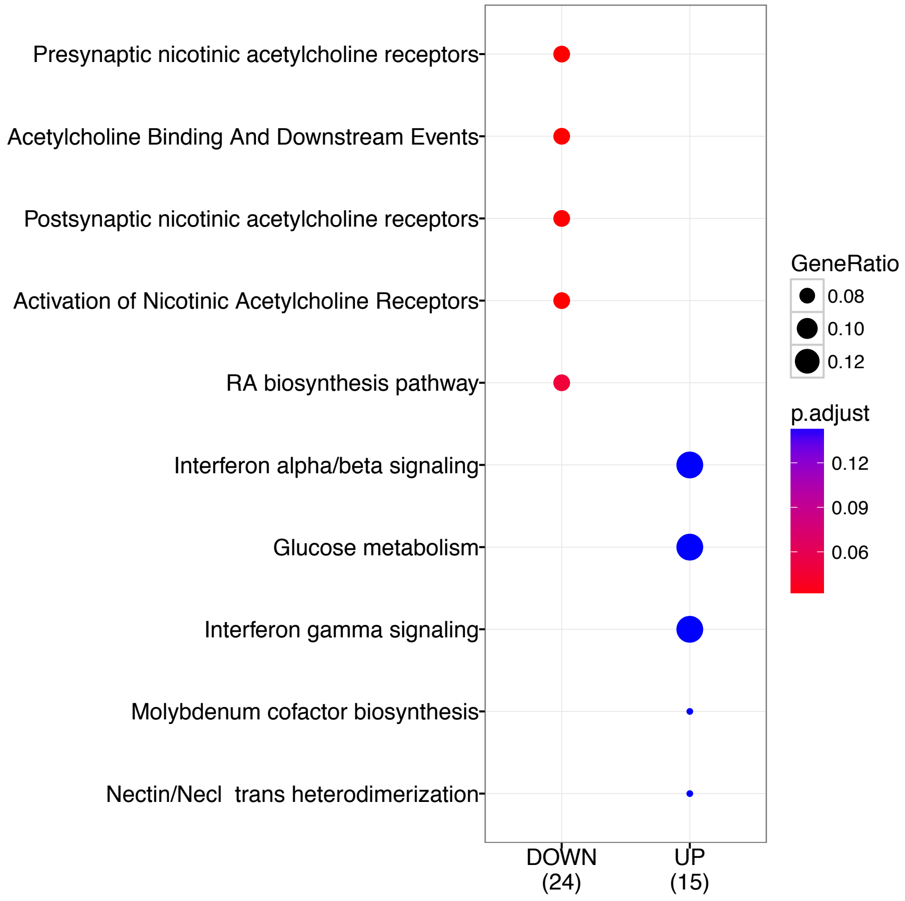
Presynaptic nicotinic acetylcholine receptors
Acetylcholine Binding And Downstream Events
Postsynaptic nicotinic acetylcholine receptors
Activation of Nicotinic Acetylcholine Receptors
RA biosynthesis pathway
Interferon alpha/beta signaling
Glucose metabolism
Interferon gamma signaling
Molybdenum cofactor biosynthesis
Nectin/Necl trans heterodimerization
DOWN
(24)
UP
(15)
GeneRatio
0.08
0.10
0.12
p.adjust
0.12
0.09
0.06

B

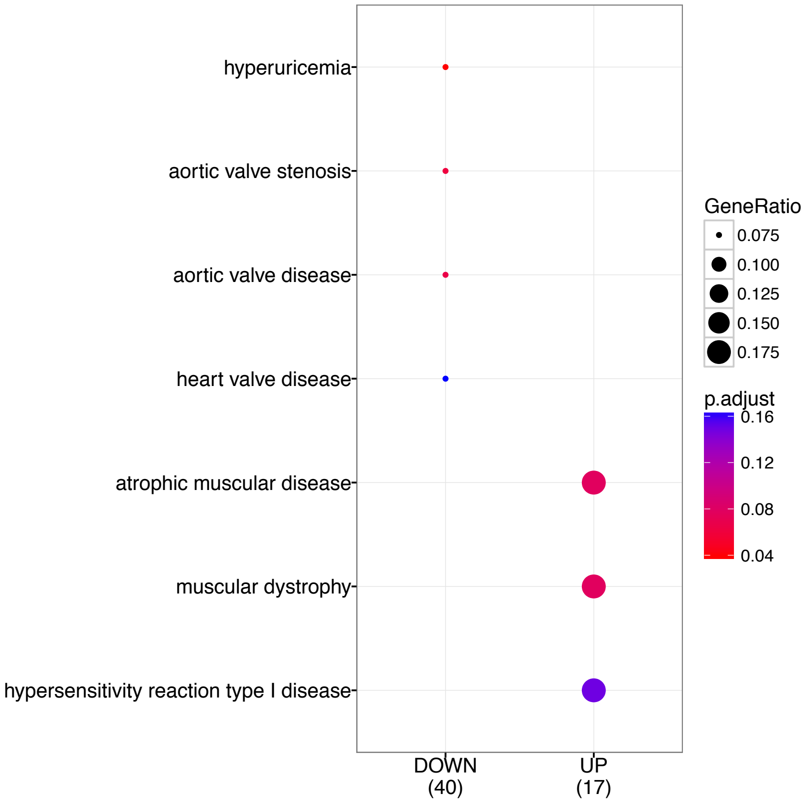

Supplementary Figure S8. Comparative study between under-expressed and over-expressed genes using ClusterProfiler method. (A) Comparative analysis based on Reactome. (B) Comparative analysis based on Disease Ontology. GeneRatio is the number of genes from a specific category. Enrichment term is represented by coloured dots (red indicates high enrichment and blue indicates low enrichment).

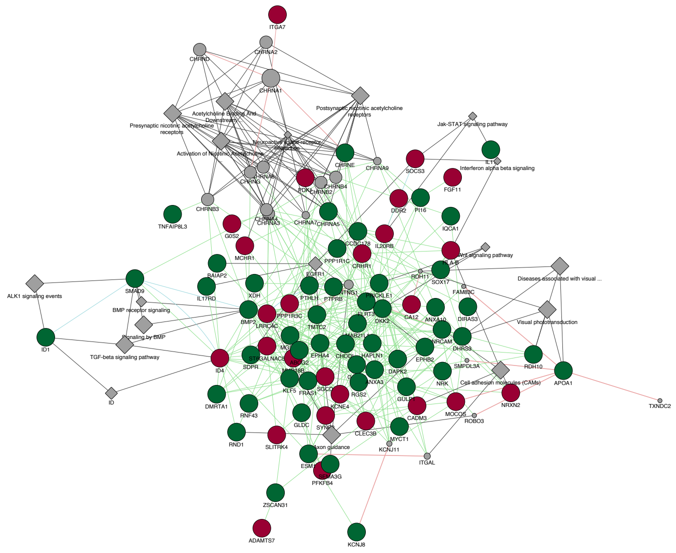

Supplementary Figure S9. GeneMANIA Network diagram. Red and green circles are query genes identified as upregulated and downregulated between S and A respectively in by RNAseq. Grey circles are extra genes connected by prediction. Rhomboid represent protein domains. Green lines represent genetic interactions. Blue lines represents pathway database

analysis. Grey lines represent gene-pathway associations. Pink lines represent physical interactions database analysis.

Gene ID	Symbol	FC	P_{adj}
ENSG00000165507	C10orf10	1,94	0,0006
ENSG00000152049	KCNE4	1,87	0,003
ENSG00000117069	ST6GALNAC5	1,83	0,009
ENSG00000135424	ITGA7	1,81	0,005
ENSG00000128285	MCHR1	1,76	0,02
ENSG00000174564	IL20RB	1,75	0,02
ENSG00000163815	CLEC3B	1,75	0,02
ENSG00000075643	MOCOS	1,75	0,001
ENSG00000074410	CA12	1,72	0,0006
ENSG00000174807	CD248	1,71	0,03
ENSG00000133454	MYO18B	1,70	0,04
ENSG00000179542	SLITRK4	1,65	0,06*
ENSG00000161570	CCL5	1,65	0,06*
ENSG00000148948	LRRC4C	1,65	0,06*
ENSG00000117289	TXNIP	1,64	0,04
ENSG00000123689	G0S2	1,63	0,05
ENSG00000162706	CADM3	1,63	0,07*
ENSG00000120088	CRHR1	1,62	0,07*
ENSG00000184557	SOCS3	1,61	0,03
ENSG00000176438	SYNE3	1,60	0,05
ENSG00000161958	FGF11	1,60	0,04
ENSG00000234745	HLA-B	1,59	0,06*
ENSG00000164236	ANKRD33B	1,59	0,09*
ENSG00000170624	SGCD	1,57	0,08*
ENSG00000259649	RP11-351M8.1	1,57	0,09*
ENSG00000172201	ID4	1,55	0,09*
ENSG00000119938	PPP1R3C	1,54	0,09*
ENSG00000114268	PFKFB4	1,54	0,04
ENSG00000110076	NRXN2	1,53	0,05
ENSG00000136378	ADAMTS7	1,52	0,02
ENSG00000152256	PDK1	1,52	0,03
ENSG00000112837	TBX18	1,51	0,09*
ENSG00000162733	DDR2	1,50	0,02
ENSG00000179348	GATA2	-1,51	0,09*
ENSG00000120693	SMAD9	-1,52	0,05
ENSG00000164736	SOX17	-1,52	0,09*

ENSG00000035664	DAPK2	-1,53	0,09*
ENSG00000144730	IL17RD	-1,54	0,01
ENSG00000133216	EPHB2	-1,55	0,03
ENSG00000166960	CCDC178	-1,55	0,09*
ENSG00000120279	MYCT1	-1,57	0,05
ENSG00000121039	RDH10	-1,57	0,06*
ENSG00000183578	TNFAIP8L3	-1,58	0,09*
ENSG00000145681	HAPLN1	-1,58	0,09*
ENSG00000158125	XDH	-1,59	0,09*
ENSG00000139174	PRICKLE1	-1,59	0,08*
ENSG00000182368	FAM27A	-1,59	0,09*
ENSG00000118137	APOA1	-1,60	0,07*
ENSG00000169684	CHRNA5	-1,60	0,09*
ENSG00000167644	C19orf33	-1,61	0,06*
ENSG00000116106	EPHA4	-1,61	0,05
ENSG00000125968	ID1	-1,61	0,04
ENSG00000123572	NRK	-1,61	0,09*
ENSG00000121361	KCNJ8	-1,63	0,07*
ENSG00000091129	NRCAM	-1,63	0,07*
ENSG00000175746	C15orf54	-1,64	0,05
ENSG00000172602	RND1	-1,64	0,04
ENSG00000095752	IL11	-1,64	0,07*
ENSG00000175866	BAIAP2	-1,65	0,01
ENSG00000127329	PTPRB	-1,65	0,06*
ENSG00000144366	GULP1	-1,66	0,005
ENSG00000143140	GJA5	-1,66	0,04
ENSG00000176399	DMRTA1	-1,68	0,05
ENSG00000168497	SDPR	-1,68	0,02
ENSG00000010319	SEMA3G	-1,69	0,02
ENSG00000254656	RTL1	-1,69	0,03
ENSG00000178445	GLDC	-1,69	0,02
ENSG00000162496	DHRS3	-1,70	0,04
ENSG00000102554	KLF5	-1,70	0,003
ENSG00000172159	FRMD3	-1,70	0,04
ENSG00000164283	ESM1	-1,71	0,03
ENSG00000235109	ZSCAN31	-1,72	0,01
ENSG00000132321	IQCA1	-1,72	0,02
ENSG00000008394	MGST1	-1,72	0,002
ENSG00000164530	PI16	-1,73	0,01
ENSG00000150722	PPP1R1C	-1,73	0,03
ENSG00000116741	RGS2	-1,74	0,02
ENSG00000180660	MAB21L1	-1,76	0,02
ENSG00000155011	DKK2	-1,77	0,01
ENSG00000126950	TMEM35	-1,81	0,02
ENSG00000179104	TMTC2	-1,81	0,004
ENSG00000118777	ABCG2	-1,85	0,01
ENSG00000125848	FLRT3	-1,87	0,007
ENSG00000108375	RNF43	-1,90	0,003

ENSG00000125845	BMP2	-1,90	0,005
ENSG00000138759	FRAS1	-1,90	0,005
ENSG00000108556	CHRNE	-1,91	0,005
ENSG00000163273	NPPC	-1,93	0,003
ENSG00000162595	DIRAS3	-1,93	0,003
ENSG00000109511	ANXA10	-1,93	0,003
ENSG00000154645	CHODL	-2,16	0,0003
ENSG00000138772	ANXA3	-2,23	0,0001
ENSG00000087494	PTHLH	-2,50	1,04E-07

Supplementary Table S1. Differential gene expression between S and A. Fold Change (FC) valued is obtained by DeSeq2 method and p adjusted value (P_{adj}) was calculated using the False Discovery Rate (FDR) method. Significant P_{adj} value was considered $P_{adj} \leq 0.05$. *Genes showing a trend towards significance but without reaching it ($P_{adj} \leq 0.09$)

Isoform id	Gene id	Symbol	FC	*P* value	P_{adj}
ENST00000358321	ENSG00000099994	SUSD2	11,61	5,00E-05	0,01
ENST00000260227	ENSG00000137673	MMP7	9,25	5,00E-05	0,01
ENST00000506608	ENSG00000185149	NPY2R	8,39	0,0002	0,04
ENST00000264613	ENSG00000047457	CP	7,90	0,0002	0,04
ENST00000374975	ENSG00000198502	HLA-DRB5	5,72	5,00E-05	0,01
ENST00000477717	ENSG00000117069	ST6GALNAC5	4,84	5,00E-05	0,01
ENST00000075322	ENSG00000136960	ENPP2	4,59	5,00E-05	0,01
ENST00000296130	ENSG00000163815	CLEC3B	4,37	5,00E-05	0,01
ENST00000394887	ENSG00000196616	ADH1B	4,07	5,00E-05	0,01
ENST00000259486	ENSG00000136960	ENPP2	3,88	5,00E-05	0,01
ENST00000013222	ENSG00000241644	INMT	3,84	5,00E-05	0,01
ENST00000393078	ENSG00000196136	SERPINA3	3,57	5,00E-05	0,01
ENST00000368125	ENSG00000162706	CADM3	3,53	0,00015	0,03
ENST00000296506	ENSG00000164106	SCRG1	3,43	5,00E-05	0,01
ENST00000256861	ENSG00000123243	ITIH5	3,42	0,00015	0,03
ENST00000294728	ENSG00000162692	VCAM1	3,38	5,00E-05	0,01
ENST00000274938	ENSG00000146197	SCUBE3	3,30	5,00E-05	0,01
ENST00000167586	ENSG00000186847	KRT14	3,06	5,00E-05	0,01
ENST00000374737	ENSG00000155659	VSIG4	3	0,00015	0,03
ENST00000372330	ENSG00000100985	MMP9	2,82	5,00E-05	0,01
ENST00000447544	ENSG00000135218	CD36	2,65	5,00E-05	0,01
ENST00000298295	ENSG00000165507	C10orf10	2,50	5,00E-05	0,01
ENST00000604125	ENSG00000152049	KCNE4	2,48	5,00E-05	0,01
ENST00000542220	ENSG00000159403	C1R	2,42	5,00E-05	0,01
ENST00000553804	ENSG00000135424	ITGA7	2,38	5,00E-05	0,01
ENST00000367662	ENSG00000116183	PAPPA2	2,34	0,00015	0,03
ENST00000006053	ENSG00000006210	CX3CL1	2,29	5,00E-05	0,01

ENST00000361673	ENSG00000198796	ALPK2	2,20	5,00E-05	0,01
ENST00000437043	ENSG00000164308	ERAP2	2,18	0,0001	0,02
ENST00000339732	ENSG00000131386	GALNT15	2,15	0,0002	0,04
ENST00000284322	ENSG00000154175	ABI3BP	2,12	5,00E-05	0,01
ENST00000261799	ENSG00000113721	PDGFRB	2,10	5,00E-05	0,01
ENST00000369317	ENSG00000117289	TXNIP	2,07	5,00E-05	0,01
ENST00000435422	ENSG00000170624	SGCD	2,06	5,00E-05	0,01
ENST00000345517	ENSG00000163017	ACTG2	2	0,00015	0,03
ENST00000257435	ENSG00000134986	NREP	1,99	5,00E-05	0,01
ENST00000429713	ENSG00000172403	SYNPO2	1,99	5,00E-05	0,01
ENST00000412585	ENSG00000234745	HLA-B	1,98	5,00E-05	0,01
ENST00000307365	ENSG00000168209	DDIT4	1,96	5,00E-05	0,01
ENST00000261326	ENSG00000075643	MOCOS	1,95	5,00E-05	0,01
ENST00000178638	ENSG00000074410	CA12	1,94	5,00E-05	0,01
ENST00000254722	ENSG00000132386	SERPINF1	1,93	5,00E-05	0,01
ENST00000335174	ENSG00000186352	ANKRD37	1,90	0,0002	0,04
ENST00000378700	ENSG00000172201	ID4	1,89	5,00E-05	0,01
ENST00000294507	ENSG00000162511	LAPTM5	1,89	5,00E-05	0,01
ENST00000540010	ENSG00000056558	TRAF1	1,89	5,00E-05	0,01
ENST00000330871	ENSG00000184557	SOCS3	1,88	5,00E-05	0,01
ENST00000344366	ENSG00000074410	CA12	1,87	5,00E-05	0,01
ENST00000238994	ENSG00000119938	PPP1R3C	1,86	5,00E-05	0,01
ENST00000217233	ENSG00000101255	TRIB3	1,84	5,00E-05	0,01
ENST00000327299	ENSG00000162433	AK4	1,83	0,0002	0,04
ENST00000328916	ENSG00000182326	C1S	1,79	0,0001	0,02
ENST00000323076	ENSG00000136167	LCP1	1,76	5,00E-05	0,01
ENST00000157600	ENSG00000071282	LMCD1	1,75	0,0001	0,02
ENST00000297848	ENSG00000187955	COL14A1	1,73	5,00E-05	0,01
ENST00000369663	ENSG00000112837	TBX18	1,73	5,00E-05	0,01
ENST00000367288	ENSG00000163431	LMOD1	1,72	5,00E-05	0,01
ENST00000338981	ENSG00000114374	USP9Y	1,70	5,00E-05	0,01
ENST00000566620	ENSG00000140945	CDH13	1,68	5,00E-05	0,01
ENST00000394308	ENSG00000070669	ASNS	1,65	0,0001	0,02
ENST00000402744	ENSG00000109472	CPE	1,65	5,00E-05	0,01
ENST00000367922	ENSG00000162733	DDR2	1,64	5,00E-05	0,01
ENST00000220244	ENSG00000103888	KIAA1199	1,62	0,0002	0,04
ENST00000380250	ENSG00000073910	FRY	1,58	0,00015	0,03
ENST00000409652	ENSG00000221963	APOL6	1,56	0,00015	0,03
ENST00000396680	ENSG00000128272	ATF4	1,55	0,00015	0,03
ENST00000372764	ENSG00000122861	PLAU	1,55	0,0001	0,02
ENST00000375377	ENSG00000165757	KIAA1462	1,54	0,00015	0,03
ENST00000263734	ENSG00000116016	EPAS1	1,53	5,00E-05	0,01
ENST00000295992	ENSG00000163710	PCOLCE2	1,53	5,00E-05	0,01
ENST00000376588	ENSG00000135069	PSAT1	1,53	0,0002	0,04
ENST00000274276	ENSG00000145623	OSMR	1,50	0,0002	0,04
ENST00000358432	ENSG00000142627	EPHA2	-1,50	5,00E-05	0,01
ENST00000260630	ENSG00000138061	CYP1B1	-1,53	0,00015	0,03
ENST00000229416	ENSG00000001084	GCLC	-1,56	0,0002	0,04

ENST00000367466	ENSG00000116711	PLA2G4A	-1,56	0,0001	0,02
ENST00000005257	ENSG00000006451	RALA	-1,56	0,0001	0,02
ENST00000245185	ENSG00000125148	MT2A	-1,57	0,0001	0,02
ENST00000361441	ENSG00000244509	APOBEC3C	-1,58	0,0001	0,02
ENST00000381434	ENSG00000137033	IL33	-1,62	0,0001	0,02
ENST00000272542	ENSG00000144136	SLC20A1	-1,63	5,00E-05	0,01
ENST00000371697	ENSG00000148677	ANKRD1	-1,64	0,00015	0,03
ENST00000248598	ENSG00000127951	FGL2	-1,67	5,00E-05	0,01
ENST00000315711	ENSG00000091972	CD200	-1,68	0,0002	0,04
ENST00000222543	ENSG00000105825	TFPI2	-1,69	5,00E-05	0,01
ENST00000237305	ENSG00000118515	SGK1	-1,72	5,00E-05	0,01
ENST00000367468	ENSG00000073756	PTGS2	-1,77	5,00E-05	0,01
ENST00000258888	ENSG00000136383	ALPK3	-1,78	5,00E-05	0,01
ENST00000426693	ENSG00000164619	BMPER	-1,81	0,0002	0,04
ENST00000379375	ENSG00000078401	EDN1	-1,86	5,00E-05	0,01
ENST00000276925	ENSG00000147883	CDKN2B	-1,91	5,00E-05	0,01
ENST00000377861	ENSG00000184226	PCDH9	-1,91	5,00E-05	0,01
ENST00000265080	ENSG00000113319	RASGRF2	-1,91	5,00E-05	0,01
ENST00000377103	ENSG00000178726	THBD	-1,93	5,00E-05	0,01
ENST00000376112	ENSG00000125968	ID1	-1,99	5,00E-05	0,01
ENST00000252593	ENSG00000130303	BST2	-2,03	5,00E-05	0,01
ENST00000396210	ENSG00000008394	MGST1	-2,04	5,00E-05	0,01
ENST00000330688	ENSG00000077092	RARB	-2,04	5,00E-05	0,01
ENST00000310613	ENSG00000173597	SULT1B1	-2,09	0,0001	0,02
ENST00000327536	ENSG00000183578	TNFAIP8L3	-2,09	5,00E-05	0,01
ENST00000274341	ENSG00000145681	HAPLN1	-2,10	5,00E-05	0,01
ENST00000304141	ENSG00000168497	SDPR	-2,10	5,00E-05	0,01
ENST00000262259	ENSG00000105497	ZNF175	-2,15	5,00E-05	0,01
ENST00000306773	ENSG00000171246	NPTX1	-2,21	5,00E-05	0,01
ENST00000222482	ENSG00000128510	CPA4	-2,27	5,00E-05	0,01
ENST00000377474	ENSG00000178695	KCTD12	-2,35	5,00E-05	0,01
ENST00000321196	ENSG00000179104	TMTC2	-2,35	5,00E-05	0,01
ENST00000315274	ENSG00000196611	MMP1	-2,40	0,00015	0,03
ENST00000444842	ENSG00000082482	KCNK2	-2,43	5,00E-05	0,01
ENST00000286614	ENSG00000156103	MMP16	-2,43	5,00E-05	0,01
ENST00000376468	ENSG00000120937	NPPB	-2,43	5,00E-05	0,01
ENST00000235382	ENSG00000116741	RGS2	-2,45	5,00E-05	0,01
ENST00000546561	ENSG00000127324	TSPAN8	-2,54	5,00E-05	0,01
ENST00000541194	ENSG00000128567	PODXL	-2,59	5,00E-05	0,01
ENST00000247829	ENSG00000127324	TSPAN8	-2,59	5,00E-05	0,01
ENST00000232892	ENSG00000114771	AADAC	-2,69	5,00E-05	0,01
ENST00000297785	ENSG00000165092	ALDH1A1	-2,72	5,00E-05	0,01
ENST00000591504	ENSG00000166510	CCDC68	-2,74	5,00E-05	0,01
ENST00000307407	ENSG00000169429	IL8	-2,82	5,00E-05	0,01
ENST00000284136	ENSG00000153993	SEMA3D	-2,85	5,00E-05	0,01
ENST00000537928	ENSG00000128567	PODXL	-2,86	0,0002	0,04
ENST00000376223	ENSG00000162496	DHRS3	-2,87	5,00E-05	0,01
ENST00000304195	ENSG00000172159	FRMD3	-3	0,00015	0,03

ENST00000359299	ENSG00000109511	ANXA10	-3,13	5,00E-05	0,01
ENST00000355754	ENSG00000162654	GBP4	-3,13	5,00E-05	0,01
ENST00000372930	ENSG00000126950	TMEM35	-3,22	5,00E-05	0,01
ENST00000296575	ENSG00000164161	HHIP	-3,48	5,00E-05	0,01
ENST00000326245	ENSG00000179914	ITLN1	-3,49	0,0001	0,02
ENST00000299502	ENSG00000197632	SERPINB2	-3,50	5,00E-05	0,01
ENST00000261292	ENSG00000101670	LIPG	-3,65	5,00E-05	0,01
ENST00000237612	ENSG00000118777	ABCG2	-3,81	5,00E-05	0,01
ENST00000397463	ENSG00000150551	LYPD1	-3,98	5,00E-05	0,01
ENST00000381405	ENSG00000164283	ESM1	-4,14	5,00E-05	0,01
ENST00000295461	ENSG00000163293	NIPAL1	-4,22	5,00E-05	0,01
ENST00000261405	ENSG00000110799	VWF	-4,25	5,00E-05	0,01
ENST00000378827	ENSG00000125845	BMP2	-4,27	5,00E-05	0,01
ENST00000263174	ENSG00000099260	PALMD	-4,36	5,00E-05	0,01
ENST00000395201	ENSG00000162595	DIRAS3	-4,39	5,00E-05	0,01
ENST00000271348	ENSG00000143140	GJA5	-4,86	5,00E-05	0,01
ENST00000409852	ENSG00000163273	NPPC	-4,93	5,00E-05	0,01
ENST00000282849	ENSG00000140873	ADAMTS18	-6,48	5,00E-05	0,01
ENST00000297316	ENSG00000164736	SOX17	-6,48	5,00E-05	0,01
ENST00000373674	ENSG00000164530	PI16	-8,28	5,00E-05	0,01

Supplementary Table S2. Analysis of differential isoform expression between S and A using Cufflinks method. Fold Change (FC) valued is obtained by DeSeq2 method and p adjusted value (P_{adj}) was calculated applying the FDR. Significant P_{adj} value was considered $P_{adj} \leq 0.05$.

Category	ID	Name	q-value	Hit Count	Hit in Query List
GO: Biological Process	GO:0072359	circulatory system development	7,91E-06	21	EPHB2,TBX18,BMP2,PTPRB,NRCAM,DHRS3,PRICKLE1,PI16,SGCD,KLF5,ANXA3,KCNJ8,SOCS3,GATA2,SOX17,ID1,XDH,ESM1,FLRT3,MYO18B,ITGA7
GO: Biological Process	GO:0072358	cardiovascular system development	7,91E-06	21	EPHB2,TBX18,BMP2,PTPRB,NRCAM,DHRS3,PRICKLE1,PI16,SGCD,KLF5,ANXA3,KCNJ8,SOCS3,GATA2,SOX17,ID1,XDH,ESM1,FLRT3,MYO18B,ITGA7
GO: Biological Process	GO:0009888	tissue development	6,90E-05	27	TBX18,BMP2,NPPC,FRAS1,ADAMTS7,PRICKLE1,PI16,SGCD,KLF5,NRK,DDR2,SOCS3,RDH10,GATA2,SOX17,ID1,ID4,XDH,CLEC3B,FLRT3,SEMA3G,MYO18B,ITGA7,PTHLH,CRHR1,EPHA4,SMAD9
GO: Biological Process	GO:0045595	regulation of cell differentiation	1,40E-04	24	EPHB2,BMP2,NPPC,CHODL,NRCAM,HLA-B,ADAMTS7,PRICKLE1,PI16,KLF5,DDR2,SOCS3,GATA2,SOX17,ID1,ID4,XDH,APOA1,SEMA3G,LRRC4C,BAIAP2,PTHLH,EPHA4,SMAD9
GO: Biological Process	GO:2000026	regulation of multicellular organismal development	1,40E-04	25	EPHB2,TBX18,BMP2,CHODL,NRCAM,HLAB,ADAMTS7,PRICKLE1,PI16,ANXA3,NRK,DDR2,SLITRK4,GATA2,SOX17,ID1,ID4,XDH,APOA1,FLRT3,SEMA3G,LRRC4C,BAIAP2,PTHLH,EPHA4
GO: Biological Process	GO:0001655	urogenital system development	2,28E-04	11	EPHB2,TBX18,BMP2,FRAS1,KCNJ8,RDH10,GATA2,SOX17,ID4,EPHA4,SMAD9

GO: Biological Process	GO:0001568	blood vessel development	4,59E-04	14	EPHB2,PTPRB,NRCAM,PRICKLE1,KLF5,ANXA3,SOCS3,GATA2,SOX17,ID1,XDH,ESM1,MYO18B,ITGA7
GO: Biological Process	GO:0001944	vasculature development	6,29E-04	14	EPHB2,PTPRB,NRCAM,PRICKLE1,KLF5,ANXA3,SOCS3,GATA2,SOX17,ID1,XDH,ESM1,MYO18B,ITGA7
GO: Biological Process	GO:0045596	negative regulation of cell differentiation	6,45E-04	14	EPHB2,BMP2,NPPC,ADAMTS7,PRICKLE1,PI16,GATA2,SOX17,ID1,ID4,XDH,SEMA3G,PTHLH,EPHA4
GO: Biological Process	GO:0051241	negative regulation of multicellular organismal process	6,45E-04	18	EPHB2,TBX18,BMP2,NPPC,ADAMTS7,PRICKLE1,PI16,GATA2,SOX17,ID1,ID4,IL20RB,XDH,APOA1,SEMA3G,RGS2,PTHLH,EPHA4
GO: Biological Process	GO:2000726	negative regulation of cardiac muscle cell differentiation	1,18E-03	3	BMP2,PRICKLE1,PI16
GO: Biological Process	GO:0048514	blood vessel morphogenesis	1,44E-03	12	EPHB2,PTPRB,NRCAM,KLF5,ANXA3,GATA2,SOX17,ID1,XDH,ESM1,MYO18B,ITGA7
Drug	D010100	Oxygen	1,76E-03	23	CA12,PPP1R3C,IL11,KCNE4,RND1,PTPRB,MCHR1,MGST1,DHRS3,PDK1,NRK,G0S2,SOCS3,XDH,TMTC2,APOA1,FGF11,CLEC3B,ABCG2,FLRT3,PFKFB4,FRMD3,HAPLN1
Drug	D002737	Chloroprene	1,82E-03	20	PPP1R3C,RND1,NRCAM,KLF5,MYCT1,G0S2,DDR2,GATA2,SOX17,ID4,XDH,ESM1,TMTC2,ABCG2,FLRT3,BAIAP2,RGS2,RNF43,PTHLH,EPHA4
GO: Biological Process	GO:0051093	negative regulation of developmental process	2,23E-03	15	EPHB2,TBX18,BMP2,NPPC,ADAMTS7,PRICKLE1,PI16,GATA2,SOX17,ID1,ID4,XDH,SEMA3G,PTHLH,EPHA4

GO: Biological Process	GO:0022603	regulation of anatomical structure morphogenesis	2,23E-03	16	EPHB2,TBX18,BMP2,SYNE3,NRCAM,ANXA3,GATA2,SOX17,ID1,XDH,APOA1,SEMA3G,LRRC4C,BAIAP2,ITGA7,EPHA4
GO: Biological Process	GO:0007507	heart development	2,23E-03	12	TBX18,BMP2,DHRS3,PRICKLE1,PI16,SGCD,KCNJ8,GATA2,SOX17,ID1,FLRT3,MYO18B
Drug	C024746	tobacco tar	2,69E-03	8	FRAS1,NRCAM,PRICKLE1,GLDC,ANXA10,ESM1,DMRTA1,RGS2
GO: Biological Process	GO:0051094	positive regulation of developmental process	2,72E-03	18	EPHB2,TBX18,BMP2,NPPC,NRCAM,KLF5,ANXA3,DDR2,SLITRK4,SOCS3,GATA2,SOX17,ID4,APOA1,FLRT3,BAIAP2,EPHA4,SMAD9
GO: Biological Process	GO:0048585	negative regulation of response to stimulus	4,05E-03	19	TBX18,BMP2,DHRS3,HLA-B,PRICKLE1,SOCS3,GATA2,SOX17,IL20RB,XDH,APOA1,FLRT3,SEMA3G,LRRC4C,RGS2,RNF43,CRHR1,DKK2,EPHA4
GO: Biological Process	GO:0002009	morphogenesis of an epithelium	4,32E-03	11	TBX18,BMP2,FRAS1,PRICKLE1,SOCS3,RDH10,SOX17,ID4,FLRT3,PTHLH,EPHA4
GO: Biological Process	GO:0010769	regulation of cell morphogenesis involved in differentiation	6,16E-03	9	EPHB2,BMP2,NRCAM,ID1,APOA1,SEMA3G,LRRC4C,BAIAP2,EPHA4
Drug	CID005282222	xanthoxin	6,22E-03	3	DHRS3,SDPR,MOCOS
Drug	C089730	rosiglitazone	6,22E-03	20	PPP1R3C,BMP2,RND1,MGST1,DHRS3,PDK1,KLF5,ANXA3,KCNJ8,G0S2,C10orf10,SLITRK4,SOCS3,ANXA10,RDH10,ID1,ID4,XDH,CLEC3B,RGS2
GO: Biological Process	GO:0022604	regulation of cell morphogenesis	7,76E-03	11	EPHB2,BMP2,SYNE3,NRCAM,ID1,APOA1,SEMA3G,LRRC4C,BAIAP2,ITGA

					7,EPHA4
Gene Family	ANXA	Annexins	8,94E-03	2	ANXA3,ANXA10
Gene Family	IL	Interleukins and interleukin receptors	8,94E-03	3	IL11,IL20RB,IL17RD
GO: Biological Process	GO:0072001	renal system development	9,14E-03	8	TBX18,BMP2,FRAS1,KCNJ8,RDH10,SOX17,EPHA4,SMAD9
Drug	CID000093003	6-chloropurine riboside	1,08E-02	3	ID1,XDH,MOCOS
Drug	CID006437077	AC1O5MYK	1,08E-02	5	PPP1R3C,TNFAIP8L3,MGST1,NRK,PPP1R1C
Drug	CID000002524	1-alpha-25-dihydroxycholecalciferol	1,08E-02	14	CA12,IL11,BMP2,TNFAIP8L3,MGST1,SOCS3,GATA2,ID1,PPP1R1C,TMTC2,BAIAP2,ITGA7,RNF43,PTHLH
GO: Biological Process	GO:0045597	positive regulation of cell differentiation	1,10E-02	14	EPHB2,BMP2,NPPC,NRCAM,KLF5,DDR2,SOCS3,GATA2,SOX17,ID4,APOA1,BAIAP2,EPHA4,SMAD9
GO: Molecular Function	GO:0052650	NADP-retinol dehydrogenase activity	1,15E-02	2	DHRS3,RDH10
GO: Molecular Function	GO:0004745	retinol dehydrogenase activity	1,15E-02	3	BMP2,DHRS3,RDH10
GO: Molecular Function	GO:0042802	identical protein binding	1,15E-02	17	EPHB2,CADM3,TBX18,BMP2,NPPC,MGST1,PDK1,GLDC,DAPK2,ID1,XDH,APOA1,DMRTA1,ABCG2,FLRT3,BAIAP2,EPHA4
GO: Molecular Function	GO:0005102	receptor binding	1,15E-02	19	EPHB2,IL11,CADM3,BMP2,NPPC,RND1,MCHR1,HLA-B,NRXN2,KCNJ8,ESM1,APOA1,FGF11,FLRT3,SEMA3G,ITGA7,RNF43,PTHLH,EPHA4
GO: Biological Process	GO:0007413	axonal fasciculation	1,21E-02	3	EPHB2,NRCAM,EPHA4

GO: Biological Process	GO:0060284	regulation of cell development	1,21E-02	14	EPHB2,BMP2,NPPC,CHODL,NRCAM,PI16,GATA2,ID1,ID4,APOA1,SEMA3G,LRRC4C,BAIAP2,EPHA4
GO: Biological Process	GO:0060429	epithelium development	1,21E-02	16	TBX18,BMP2,FRAS1,PRICKLE1,KLF5,SOCS3,RDH10,SOX17,ID1,ID4,XDH,FLRT3,PTHLH,CRHR1,EPHA4,SMAD9
GO: Biological Process	GO:0055026	negative regulation of cardiac muscle tissue development	1,31E-02	3	BMP2,PRICKLE1,PI16
GO: Biological Process	GO:0051960	regulation of nervous system development	1,32E-02	13	EPHB2,BMP2,CHODL,NRCAM,SLITRK4,GATA2,ID1,ID4,FLRT3,SEMA3G,LRRC4C,BAIAP2,EPHA4
GO: Biological Process	GO:0045664	regulation of neuron differentiation	1,36E-02	11	EPHB2,BMP2,CHODL,NRCAM,GATA2,ID1,ID4,SEMA3G,LRRC4C,BAIAP2,EPHA4
GO: Molecular Function	GO:0030151	molybdenum ion binding	1,49E-02	2	XDH,MOCOS
GO: Molecular Function	GO:0045499	chemorepellent activity	1,49E-02	3	APOA1,FLRT3,SEMA3G
GO: Molecular Function	GO:0046983	protein dimerization activity	1,49E-02	16	CADM3,TBX18,BMP2,NPPC,MGST1,PDK1,GLDC,SOX17,ID1,ID4,XDH,DMRTA1,ABCG2,FLRT3,ITGA7,CHRNA5
GO: Biological Process	GO:0048598	embryonic morphogenesis	1,51E-02	11	EPHB2,TBX18,FRAS1,PRICKLE1,SOCS3,RDH10,GATA2,SOX17,APOA1,FLRT3,ITGA7
GO: Biological Process	GO:0009790	embryo development	1,51E-02	15	EPHB2,TBX18,BMP2,FRAS1,PRICKLE1,NRK,SOCS3,RDH10,GATA2,SOX17,APOA1,ABCG2,FLRT3,MYO18B,ITGA7
GO: Biological Process	GO:0023057	negative regulation of signaling	1,51E-02	16	IL11,TBX18,BMP2,DHRS3,PRICKLE1,SOCS3,GATA2,SOX17,XDH,APOA1,FLRT3,LRRC4C,RGS2,RNF43,CRHR1,DKK2

GO: Molecular Function	GO:0042803	protein homodimerization activity	1,53E-02	12	CADM3,TBX18,BMP2,NPPC,MGST1,PDK1,GLDC,ID1,XDH,DMRTA1,ABCG2,FLRT3
GO: Cellular Component	GO:0005887	integral component of plasma membrane	1,56E-02	19	EPHB2,CADM3,BMP2,KCNE4,PTPRB,MCHR1,NRCAM,HLA-B,SGCD,KCNJ8,DDR2,FLRT3,ITGA7,IL17RD,RNF43,CRHR1,CHRNA5,CHRNE,EPHA4
GO: Cellular Component	GO:0031226	intrinsic component of plasma membrane	1,56E-02	19	EPHB2,CADM3,BMP2,KCNE4,PTPRB,MCHR1,NRCAM,HLA-B,SGCD,KCNJ8,DDR2,FLRT3,ITGA7,IL17RD,RNF43,CRHR1,CHRNA5,CHRNE,EPHA4
GO: Biological Process	GO:0003308	negative regulation of Wnt signaling pathway involved in heart development	1,60E-02	2	BMP2,SOX17
GO: Biological Process	GO:0010648	negative regulation of cell communication	1,60E-02	16	IL11,TBX18,BMP2,DHRS3,PRICKLE1,SOCS3,GATA2,SOX17,XDH,APOA1,FLRT3,LRRC4C,RGS2,RNF43,CRHR1,DKK2
GO: Biological Process	GO:2000725	regulation of cardiac muscle cell differentiation	1,61E-02	3	BMP2,PRICKLE1,PI16
GO: Biological Process	GO:0045669	positive regulation of osteoblast differentiation	1,61E-02	4	BMP2,NPPC,DDR2,ID4
GO: Biological Process	GO:0045667	regulation of osteoblast differentiation	1,61E-02	5	BMP2,NPPC,DDR2,ID1,ID4
GO: Biological Process	GO:0001525	angiogenesis	1,61E-02	9	EPHB2,PTPRB,NRCAM,KLF5,ANXA3,GATA2,SOX17,ID1,ESM1

GO: Biological Process	GO:0051174	regulation of phosphorus metabolic process	1,61E-02	20	IL11,BMP2,NPPC,TNFAIP8L3,PDK1,NRK,DDR2,SOCS3,ID1,PPP1R1C,XDH,APOA1,FLRT3,LRRC4C,IL17RD,RGS2,PTHLH,CRHR1,DIRAS3,EPHA4
GO: Biological Process	GO:0045995	regulation of embryonic development	1,67E-02	5	TBX18,NRK,GATA2,SOX17,APOA1
GO: Biological Process	GO:0035295	tube development	1,68E-02	11	TBX18,BMP2,PRICKLE1,KLF5,RDH10,GATA2,SOX17,ID1,PTHLH,EPHA4,SMAD9
GO: Biological Process	GO:0030178	negative regulation of Wnt signaling pathway	1,68E-02	6	TBX18,BMP2,PRICKLE1,SOX17,RNF43,DKK2
GO: Biological Process	GO:0048729	tissue morphogenesis	1,73E-02	11	TBX18,BMP2,FRAS1,PRICKLE1,SOCS3,RDH10,SOX17,ID4,FLRT3,PTHLH,EPHA4
GO: Biological Process	GO:0051154	negative regulation of striated muscle cell differentiation	1,79E-02	3	BMP2,PRICKLE1,PI16
GO: Biological Process	GO:0030278	regulation of ossification	1,79E-02	6	BMP2,NPPC,DHRS3,DDR2,ID1,ID4
Drug	D012822	Silicon Dioxide	1,93E-02	21	IL11,BMP2,NPPC,FRAS1,PTPRB,PI16,KCNJ8,G0S2,SLITRK4,SOCS3,XDH,ESM1,DMRTA1,CLEC3B,ABCG2,FLRT3,SEMA3G,LRRC4C,RGS2,DIRAS3,HAPLN1
GO: Biological Process	GO:0048710	regulation of astrocyte differentiation	1,94E-02	3	BMP2,ID4,EPHA4
GO: Biological Process	GO:0006468	protein phosphorylation	2,00E-02	20	EPHB2,IL11,BMP2,TNFAIP8L3,PDK1,DAPK2,NRK,DDR2,SOCS3,ID1,PPP1R1C,XDH,APOA1,FLRT3,LRRC4C,IL17RD,RGS2,DIRAS3,EPHA4,SMAD9
GO: Biological Process	GO:0048738	cardiac muscle tissue development	2,01E-02	6	TBX18,BMP2,PRICKLE1,PI16,SGCD,MYO18B

GO: Biological Process	GO:0014706	striated muscle tissue development	2,01E-02	8	TBX18,BMP2,PRICKLE1,PI16,SGCD,KLF5,SOX17,MYO18B
GO: Biological Process	GO:0040007	growth	2,01E-02	14	BMP2,NPPC,NRCAM,PI16,NRK,DDR2,SOCS3,RDH10,SOX17,PPP1R1C,ESM1,FLRT3,SEMA3G,PTHLH
Drug	CID003619466	rotigotine patch	2,15E-02	5	CA12,PTPRB,TNFAIP8L3,CD248,CLEC3B
GO: Biological Process	GO:0003307	regulation of Wnt signaling pathway involved in heart development	2,15E-02	2	BMP2,SOX17
GO: Biological Process	GO:0001503	ossification	2,18E-02	8	BMP2,NPPC,DHRS3,DDR2,ID1,ID4,CLEC3B,PTHLH
Drug	C015001	arsenite	2,18E-02	19	EPHB2,PPP1R3C,NPPC,FRAS1,DHRS3,NRXN2,PRICKLE1,SGCD,KLF5,G0S2,ST6GALNAC5,SOX17,BAIAP2,ITGA7,PTHLH,MAB21L1,SDPR,DIRAS3,FRMD3
Drug	D003907	Dexamethasone	2,18E-02	19	CA12,BMP2,NPPC,MGST1,DHRS3,NRXN2,PI16,PDK1,KLF5,ANXA3,G0S2,XDH,APOA1,CLEC3B,ABCG2,RGS2,PTHLH,SDPR,GULP1
GO: Biological Process	GO:0035051	cardiocyte differentiation	2,24E-02	5	BMP2,PRICKLE1,PI16,SOX17,MYO18B
GO: Biological Process	GO:0007517	muscle organ development	2,24E-02	8	BMP2,CHODL,PRICKLE1,PI16,SGCD,KLF5,SOX17,ITGA7
Drug	CID000129935	CGP 31608	2,25E-02	2	CD248,ID1
Drug	C032668	1-nitropyrene	2,25E-02	7	BMP2,PTPRB,G0S2,XDH,ESM1,PTHLH,MOCOS

Drug	D010126	Ozone	2,25E-02	15	NPPC,KCNE4,MGST1,NRXN2,KLF5,MYCT1,ANXA3,KCNJ8,G0S2,SOCS3,ID1,CLEC3B,ABCG2,PFKFB4,SDPR
Drug	C057693	troglitazone	2,25E-02	16	CA12,IL11,BMP2,RND1,DHRS3,ANXA3,KCNJ8,G0S2,C10orf10,ANXA10,GATA2,ID1,ID4,APOA1,ABCG2,RGS2
Drug	D008070	Lipopolysaccharides	2,25E-02	17	EPHB2,IL11,BMP2,PDK1,KCNJ8,G0S2,SOCS3,GATA2,SOX17,ID4,ESM1,APOA1,ABCG2,ITGA7,IL17RD,RGS2,CRHR1
Drug	D019833	Genistein	2,25E-02	20	CA12,PPP1R3C,BMP2,NPPC,MGST1,DHRS3,ADAMTS7,PDK1,GLDC,ANXA3,G0S2,SOCS3,ID1,ID4,XDH,ESM1,APOA1,ABCG2,PFKFB4,CHRNA5
GO: Biological Process	GO:0001649	osteoblast differentiation	2,27E-02	6	BMP2,NPPC,DDR2,ID1,ID4,PTHLH
Drug	D013755	Tetradecanoylphorbol Acetate	2,27E-02	12	IL11,BMP2,NRXN2,ANXA3,DAPK2,SOCS3,ANXA10,GATA2,SOX17,XDH,ITGA7,PTHLH
GO: Biological Process	GO:0045444	fat cell differentiation	2,33E-02	6	IL11,BMP2,KLF5,GATA2,ID4,RGS2
Drug	CID000002619	AC1L1E32	2,37E-02	2	CD248,ABCG2
Drug	C038518	acrylodan	2,37E-02	2	MGST1,APOA1
Drug	CID000005325	sulfamerazine	2,37E-02	3	MGST1,SGCD,IL17RD
Drug	MESH:D013274/D004052-M	Stomach Neoplasms affected by Diethylnitrosamine	2,37E-02	4	BMP2,ID4,APOA1,RGS2
Drug	D003609	Dactinomycin	2,37E-02	7	IL11,NPPC,SOCS3,SOX17,ID1,ID4,ABCG2
Drug	D015124	8-Bromo Cyclic Adenosine Monophosphate	2,37E-02	11	PPP1R3C,FRAS1,KCNE4,MGST1,NRCAM,DHRS3,SGCD,SOCS3,ID1,PTHLH,MOCOS

Drug	C548651	2-(1'H-indolo-3'-carbonyl)thiazole-4-carboxylic acid methyl ester	2,37E-02	12	PPP1R3C,BMP2,PTPRB,CD248,PDK1,DDR2,ID1,ID4,XDH,FLRT3,EPHA4,GULP1
Drug	D000393	Air Pollutants	2,37E-02	13	CA12,IL11,FRAS1,RND1,TNFAIP8L3,DHRS3,ANXA3,ANXA10,ID1,XDH,ABCG2,SDPR,FRMD3
Drug	CID000003003	Betamethasone-d5	2,37E-02	16	IL11,BMP2,NPPC,TNFAIP8L3,ANXA3,ANXA10,ID1,XDH,APOA1,CLEC3B,ABCG2,ITGA7,PTHLH,CRHR1,CHRNE,HAPLN1
GO: Biological Process	GO:0060537	muscle tissue development	2,38E-02	8	TBX18,BMP2,PRICKLE1,PI16,SGCD,KLF5,SOX17,MYO18B
GO: Biological Process	GO:0045602	negative regulation of endothelial cell differentiation	2,42E-02	2	ID1,XDH
GO: Biological Process	GO:0031648	protein destabilization	2,42E-02	3	BMP2,SOX17,ID1
GO: Biological Process	GO:0008038	neuron recognition	2,42E-02	3	EPHB2,NRCAM,EPHA4
GO: Biological Process	GO:0007416	synapse assembly	2,42E-02	5	EPHB2,NRCAM,NRXN2,SLITRK4,FLRT3
GO: Biological Process	GO:0000904	cell morphogenesis involved in differentiation	2,42E-02	14	EPHB2,BMP2,RND1,NRCAM,SLITRK4,SOX17,ID1,APOA1,FLRT3,SEMA3G,LRRC4C,BAIAP2,IL17RD,EPHA4
GO: Biological Process	GO:0009968	negative regulation of signal transduction	2,42E-02	14	TBX18,BMP2,DHRS3,PRICKLE1,SOCS3,GATA2,SOX17,XDH,APOA1,FLRT3,LRRC4C,RGS2,RNF43,DKK2
GO: Biological Process	GO:0019220	regulation of phosphate metabolic process	2,42E-02	19	IL11,BMP2,NPPC,TNFAIP8L3,NRK,DDR2,SOCS3,ID1,PPP1R1C,XDH,APOA1,FLRT3,LRRC4C,IL17RD,RGS2,PTHLH,CRHR1,DIRAS3,EPHA4

GO: Biological Process	GO:0048646	anatomical structure formation involved in morphogenesis	2,45E-02	15	EPHB2,TBX18,BMP2,PTPRB,NRCAM,PRICKLE1,KLF5,ANXA3,RDH10,GATA2,SOX17,ID1,ESM1,ITGA7,PTHLH
Drug	CID000057056	AC1L1M7Y	2,53E-02	4	IL11,SOCS3,RGS2,PTHLH
Disease	ctd:D002357	Cartilage Diseases	2,57E-02	2	NPPC,GATA2
Disease	ctd:D003865	Depressive Disorder, Major	2,57E-02	2	XDH,CRHR1
Disease	ctd:D001528	Behcet Syndrome	2,57E-02	2	HLA-B,APOA1
Disease	ctd:D003875	Drug Eruptions	2,57E-02	2	HLA-B,APOA1
GO: Biological Process	GO:0001501	skeletal system development	2,62E-02	9	BMP2,NPPC,DHRS3,ADAMTS7,RDH10,CLEC3B,PTHLH,HAPLN1,SMAD9
GO: Biological Process	GO:0050767	regulation of neurogenesis	2,73E-02	11	EPHB2,BMP2,CHODL,NRCAM,GATA2,ID1,ID4,SEMA3G,LRRC4C,BAIAP2,EPHA4
GO: Biological Process	GO:0060997	dendritic spine morphogenesis	2,80E-02	3	EPHB2,BAIAP2,EPHA4
GO: Biological Process	GO:0061061	muscle structure development	2,80E-02	10	TBX18,BMP2,CHODL,PRICKLE1,PI16,SGCD,KLF5,SOX17,MYO18B,ITGA7
Drug	CID000003640	AC1Q5HK6	2,86E-02	9	IL11,BMP2,TNFAIP8L3,XDH,ESM1,APOA1,PTHLH,CRHR1,CHRNE
Disease	ctd:D015179	Colorectal Neoplasms	2,90E-02	5	NRCAM,SOX17,ABCG2,RNF43,HAPLN1
Drug	CID000003413	forskolin from Coleus forskohlii	3,11E-02	13	PPP1R3C,BMP2,NPPC,TNFAIP8L3,MCHR1,KCNJ8,NRK,XDH,RGS2,PTHLH,CRHR1,CHRNE,HAPLN1
Disease	ctd:D020022	Genetic Predisposition to Disease	3,11E-02	2	HLA-B,KCNJ8
Disease	ctd:D008325	Mammary Neoplasms, Experimental	3,11E-02	4	G0S2,ID1,ID4,XDH
GO: Biological Process	GO:0090090	negative regulation of canonical Wnt signaling pathway	3,14E-02	5	TBX18,BMP2,PRICKLE1,SOX17,DKK2

Drug	D008748	Methylcholanthrene	3,26E-02	13	CA12,NRXN2,KLF5,DAPK2,SOCS3,SOX17,XDH,TMTC2,APOA1,ABCG2,BAIAP2,ITGA7,GULP1
Drug	C022909	5-methoxypsoralen	3,27E-02	2	BMP2,SMAD9
GO: Biological Process	GO:0050919	negative chemotaxis	3,37E-02	3	APOA1,FLRT3,SEMA3G
GO: Biological Process	GO:0045778	positive regulation of ossification	3,37E-02	4	BMP2,NPPC,DDR2,ID4
GO: Biological Process	GO:0072089	stem cell proliferation	3,37E-02	5	TBX18,BMP2,SOX17,ID4,RNF43
GO: Biological Process	GO:0010721	negative regulation of cell development	3,38E-02	7	EPHB2,NPPC,PI16,ID1,ID4,SEMA3G,EPHA4
Drug	D005944	Glucosamine	3,39E-02	7	IL11,BMP2,RND1,ID1,XDH,APOA1,HAPLN1
GO: Biological Process	GO:0045843	negative regulation of striated muscle tissue development	3,67E-02	3	BMP2,PRICKLE1,PI16
GO: Molecular Function	GO:0005005	transmembrane-ephrin receptor activity	3,75E-02	2	EPHB2,EPHA4
Disease	ctd:D001008	Anxiety Disorders	3,80E-02	2	CRHR1,CHRNA5
Disease	ctd:D006130	Growth Disorders	3,82E-02	2	NPPC,PTHLH
Disease	ctd:D001172	Arthritis, Rheumatoid	3,82E-02	4	ANXA3,ST6GALNAC5,ABCG2,HAPLN1
Disease	ctd:D013274	Stomach Neoplasms	3,82E-02	5	BMP2,ID4,APOA1,RGS2,RNF43
GO: Biological Process	GO:0001932	regulation of protein phosphorylation	3,85E-02	16	IL11,BMP2,TNFAIP8L3,NRK,DDR2,SOCS3,ID1,PPP1R1C,XDH,APOA1,FLRT3,LRRC4C,IL17RD,RGS2,DIRAS3,EPHA4
Drug	CID000004938	propentofylline	3,92E-02	3	XDH,EPHA4,HAPLN1
GO: Biological Process	GO:0048635	negative regulation of muscle organ development	4,07E-02	3	BMP2,PRICKLE1,PI16

Drug	D012906	Smoke	4,09E-02	12	CA12,NRCAM,PRICKLE1,GLDC,KLF5,ANXA3,ST6GALNAC5,SOCS3,IL20RB,TMTC2,ABCG2,MOCOS
GO: Biological Process	GO:0002062	chondrocyte differentiation	4,10E-02	4	BMP2,NPPC,ADAMTS7,PTHLH
GO: Biological Process	GO:0061448	connective tissue development	4,11E-02	6	BMP2,NPPC,ADAMTS7,ID4,PTHLH,SMAD9
GO: Biological Process	GO:1901862	negative regulation of muscle tissue development	4,11E-02	3	BMP2,PRICKLE1,PI16
GO: Biological Process	GO:0046426	negative regulation of JAK-STAT cascade	4,11E-02	3	SOCS3,FLRT3,LRRC4C
GO: Biological Process	GO:0003306	Wnt signaling pathway involved in heart development	4,11E-02	2	BMP2,SOX17
GO: Biological Process	GO:0034113	heterotypic cell-cell adhesion	4,16E-02	3	NRCAM,APOA1,ITGA7
GO: Biological Process	GO:0097061	dendritic spine organization	4,16E-02	3	EPHB2,BAIAP2,EPHA4
GO: Biological Process	GO:0010975	regulation of neuron projection development	4,16E-02	8	EPHB2,CHODL,NRCAM,ID1,SEMA3G,LRRC4C,BAIAP2,EPHA4
GO: Biological Process	GO:0009792	embryo development ending in birth or egg hatching	4,16E-02	10	TBX18,BMP2,PRICKLE1,NRK,SOCS3,RDH10,GATA2,SOX17,ABCG2,MYO18B
GO: Biological Process	GO:0061564	axon development	4,19E-02	11	EPHB2,RND1,NRCAM,SLITRK4,APOA1,FLRT3,SEMA3G,LRRC4C,BAIAP2,IL17RD,EPHA4
Drug	CID000005337	anti-K	4,22E-02	3	HLA-B,SGCD,NRK
GO: Biological Process	GO:0051338	regulation of transferase activity	4,25E-02	13	BMP2,TNFAIP8L3,NRK,DDR2,SOCS3,PPP1R1C,APOA1,FLRT3,LRRC4C,IL17RD,RGS2,DIRAS3,EPHA4
GO: Biological Process	GO:0048568	embryonic organ development	4,26E-02	8	EPHB2,TBX18,BMP2,NRK,SOCS3,RDH10,GATA2,SOX17

GO: Biological Process	GO:0010614	negative regulation of cardiac muscle hypertrophy	4,32E-02	2	PI16,RGS2
GO: Biological Process	GO:1900745	positive regulation of p38MAPK cascade	4,32E-02	2	BMP2,XDH
GO: Biological Process	GO:0030282	bone mineralization	4,32E-02	4	BMP2,DDR2,CLEC3B,PTHLH
GO: Biological Process	GO:0050770	regulation of axonogenesis	4,33E-02	5	EPHB2,NRCAM,SEMA3G,LRRC4C,EPHA4
GO: Biological Process	GO:0043549	regulation of kinase activity	4,33E-02	12	BMP2,TNFAIP8L3,NRK,DDR2,SOCS3,PPP1R1C,FLRT3,LRRC4C,IL17RD,RGS2,DIRAS3,EPHA4
Drug	CID005282224	abscisic aldehyde	4,36E-02	2	DHRS3,SDPR
Disease	ctd:C538231	Adenocarcinoma of lung	4,36E-02	2	DDR2,APOA1
Disease	ctd:D000022	Abortion, Spontaneous	4,36E-02	3	IL11,FLRT3,PTHLH
Disease	ctd:D001943	Breast Neoplasms	4,50E-02	6	BMP2,NRCAM,C10orf10,ABCG2,RGS2,PTHLH
Drug	CID005321959	Tn-1	4,53E-02	3	HLA-B,CD248,CLEC3B
GO: Biological Process	GO:0060348	bone development	4,60E-02	5	BMP2,NPPC,DHRS3,PTHLH,SMAD9
Drug	CID000005566	trifluoperazine	4,61E-02	7	PPP1R3C,ANXA3,ANXA10,PPP1R1C,XDH,MYO18B,CHRNE
Drug	MESH:D001943 /C014347-T	Breast Neoplasms affected by decitabine	4,61E-02	4	BMP2,NRCAM,ABCG2,RGS2
GO: Biological Process	GO:0055007	cardiac muscle cell differentiation	4,69E-02	4	BMP2,PRICKLE1,PI16,MYO18B
GO: Biological Process	GO:0060033	anatomical structure regression	4,76E-02	2	CD248,SMAD9
Drug	CID000007272	lactide	4,77E-02	3	BMP2,MOCOS,HAPLN1
Drug	CID000000338	salicylate	4,77E-02	7	TNFAIP8L3,MCHR1,SOCS3,XDH,CLEC3B,ABCG2,MOCOS
Disease	ctd:D064420	Drug-Related Side Effects and Adverse Reactions	5,01E-02	2	HLA-B,ABCG2

GO: Biological Process	GO:0014741	negative regulation of muscle hypertrophy	5,05E-02	2	PI16,RGS2
GO: Biological Process	GO:0060712	spongiotrophoblast layer development	5,05E-02	2	NRK,SOCS3
GO: Biological Process	GO:0045598	regulation of fat cell differentiation	5,05E-02	4	BMP2,KLF5,GATA2,ID4
GO: Biological Process	GO:0051216	cartilage development	5,05E-02	5	BMP2,NPPC,ADAMTS7,PTHLH,SMAD9
GO: Biological Process	GO:0001822	kidney development	5,05E-02	6	BMP2,FRAS1,KCNJ8,RDH10,EPHA4,SMAD9
GO: Biological Process	GO:0031344	regulation of cell projection organization	5,05E-02	9	EPHB2,CHODL,NRCAM,KLF5,ID1,SEMA3G,LRRC4C,BAIAP2,EPHA4
GO: Biological Process	GO:0016477	cell migration	5,05E-02	14	BMP2,NRCAM,CD248,ANXA3,DAPK2,DDR2,GATA2,SOX17,ID1,APOA1,FLRT3,SEMA3G,ITGA7,EPHA4
GO: Biological Process	GO:0000902	cell morphogenesis	5,05E-02	16	EPHB2,BMP2,RND1,SYNE3,NRCAM,SLITRK4,SOX17,ID1,APOA1,FLRT3,SEMA3G,LRRC4C,BAIAP2,ITGA7,IL17RD,EPHA4
GO: Biological Process	GO:0051240	positive regulation of multicellular organismal process	5,05E-02	16	EPHB2,TBX18,BMP2,NPPC,NRCAM,ANXA3,DDR2,SLITRK4,GATA2,SOX17,ID4,IL20RB,FLRT3,BAIAP2,RGS2,EPHA4
GO: Biological Process	GO:0031399	regulation of protein modification process	5,06E-02	18	IL11,BMP2,TNFAIP8L3,PRICKLE1,NRK,DDR2,SOCS3,GATA2,ID1,PPP1R1C,XDH,APOA1,FLRT3,LRRC4C,IL17RD,RGS2,DIRAS3,EPHA4
GO: Biological Process	GO:0007167	enzyme linked receptor protein signaling pathway	5,09E-02	14	EPHB2,BMP2,NPPC,DDR2,SOCS3,ID1,XDH,ESM1,APOA1,FLRT3,BAIAP2,IL17RD,EPHA4,SMAD9

Supplementary Table S3. Molecular enrichment associations analysis on DEG performed with Bioconductor tool. Significant annotations with q-value ≤ 0,05 are shown.

Category	ID	Name	q-value	Hit Count	Hit in Query List
Drug	D003907	Dexamethasone	8,61E-18	51	GALNT15,CA12,KCTD12,CYP1B1,AADAC,DDIT4,NPPB,PSAT1,NPPC,KRT14,DHRS3,PDGFRB,PI16,LMCD1,SGK1,ENPP2,SERPINF1,ACTG2,CP,CPE,RGS2,PTGS2,EDN1,TXNIP,BMP2,GJA5,MT2A,TRIB3,MGST1,GCLC,SLC20A1,CD36,ANKRD1,ASNS,KCNK2,PLA2G4A,PLAU,CLEC3B,ATF4,ABCG2,ALDH1A1,MMP1,BMPER,MMP9,MMP16,CX3CL1,IL33,VCAM1,SDPR,ALPK2,CDH13
Drug	D010100	Oxygen	6,51E-17	49	CA12,CYP1B1,DDIT4,NPPB,PSAT1,SYNPO2,NPTX1,DHRS3,PDGFRB,LMCD1,PODXL,SGK1,ANKRD37,ENPP2,SOCS3,ACTG2,CP,TMTC2,CPE,NREP,PTGS2,EDN1,ITIH5,HAPLN1,PPP1R3C,TXNIP,MT2A,KCNE4,MGST1,GCLC,LIPG,CD36,ANKRD1,ASNS,SERPINB2,COL14A1,AK4,PLAU,CLEC3B,ATF4,ABCG2,MMP1,MMP7,MMP9,CX3CL1,EPAS1,VCAM1,CDH13,FRMD3
GO: Biological Process	GO:0072359	circulatory system development	1,14E-11	35	CYP1B1,NPPB,NPY2R,RARB,DHRS3,PDGFRB,PI16,SGCD,ENPP2,SERPINF1,SOCS3,SOX17,ID1,ESM1,CPE,ITGA7,PTGS2,EDN1,TBX18,BMP2,GJA5,HHIP,EPHA2,CD36,ANKRD1,KCNK2,ALPK3,COL14A1,PLAU,BMPER,MMP9,CX3CL1,EPAS1,VCAM1,CDH13

GO: Biological Process	GO:0072358	cardiovascular system development	1,14E-11	35	CYP1B1,NPPB,NPY2R,RARB,DHRS3,PDGFRB,PI16,SGCD,ENPP2,SERPINF1,SOCS3,SOX17,ID1,ESM1,CPE,ITGA7,PTGS2,EDN1,TBX18,BMP2,GJA5,HHIP,EPHA2,CD36,ANKRD1,KCNK2,ALPK3,COL14A1,PLAU,BMPER,MMP9,CX3CL1,EPAS1,VCAM1,CDH13
GO: Cellular Component	GO:0005615	extracellular space	1,16E-09	37	CPA4,SERPINA3,NPPB,NPPC,ABI3BP,PI16,LMCD1,PODXL,ENPP2,HLA-DRB5,SERPINF1,ACTG2,CP,SCRG1,CPE,LCP1,EDN1,FGL2,BMP2,SEMA3D,THBD,LIPG,CD36,SERPINB2,C1R,C1S,COL14A1,PLAU,CLEC3B,MMP1,BMPER,MMP7,MMP9,CX3CL1,IL33,VCAM1,CDH13
GO: Biological Process	GO:0001525	angiogenesis	1,04E-07	20	CYP1B1,NPPB,PDGFRB,ENPP2,SERPINF1,SOX17,ID1,ESM1,PTGS2,EDN1,GJA5,HHIP,EPHA2,CD36,PLAU,BMPER,MMP9,CX3CL1,EPAS1,CDH13
Pathway	812256	TNF signaling pathway	1,74E-03	8	TRAF1,SOCS3,PTGS2,EDN1,ATF4,MMP9,CX3CL1,VCAM1
Pathway	576262	Extracellular matrix organization	1,74E-03	12	DDR2,ITGA7,HAPLN1,BMP2,ADAMTS18,COL14A1,PCOLCE2,MMP1,MMP7,MMP9,MMP16,VCAM1
GO: Cellular Component	GO:0098590	plasma membrane region	2,53E-03	20	KCTD12,DHRS3,PDGFRB,PODXL,ITLN1,DDR2,CPE,LCP1,PTGS2,HHIP,KCNE4,BST2,EPHA2,CD36,KCNK2,ATF4,ABCG2,OSMR,SDPR,CDH13

Supplementary Table S4. Bioconductor molecular enrichment associations analysis on DEI. Selected annotations illustrated are those showing significant q-value ≤ 0,003 from each category.

Patient characteristics	All	Symptomatic	Asymptomatic
Number, n	11	4	7
Years	68	68±8	70±12
Sex M/F, n	7/4	1/3	6/1
Treatment with statins	10	3	7
**Risks Factors (%)**			
Diabetes Mellitus	6	3 (75%)	3 (43%)
Dyslipidemia	10	4 (100%)	6 (86%)
Arterial hypertension	9	3 (75%)	6 (86%)
Tobacco	5	3 (75%)	2 (29%)

Supplementary Table S5. Demographic data for Basurto University Hospital cohort of additional 11 samples
